# Overview of Preclinical and Clinical Trials of Nanoparticles for the Treatment of Brain Metastases

**DOI:** 10.3390/pharmaceutics17070899

**Published:** 2025-07-11

**Authors:** Muhammad Izhar, Mohamed Al Gharyani, Ahed H. Kattaa, Juan J. Cardona, Ruchit P. Jain, Elaheh Shaghaghian, Yusuke S. Hori, Fred C. Lam, Deyaaldeen Abu Reesh, Sara C. Emrich, Louisa Ustrzynski, Armine Tayag, Maciej S. Lesniak, Steven D. Chang, David J. Park

**Affiliations:** 1 Department of Neurosurgery, Stanford University School of Medicine, Stanford, CA 94305, USA; 2 Department of Neurological Surgery, Lou and Jean Malnati Brain Tumor Institute, Robert H. Lurie Comprehensive Cancer Center, Feinberg School of Medicine, Northwestern University, Chicago, IL 60611, USA

**Keywords:** nanoparticles, nanomaterials, nanocarriers, nanomedicine, nanotherapeutics, brain metastases

## Abstract

Brain metastases (BM), which most commonly originate from lung, breast, or skin cancers, remain a major clinical challenge, with standard treatments such as stereotactic radiosurgery (SRS), surgical resection, and whole-brain radiation therapy (WBRT). The prognosis for patients with BM remains poor, with a median overall survival (OS) of just 10–16 months. Although recent advances in systemic therapies, including small molecule inhibitors, monoclonal antibodies, chemotherapeutics, and gene therapies, have demonstrated success in other malignancies, their effectiveness in central nervous system (CNS) cancers is significantly limited by poor blood–brain barrier (BBB) permeability and subtherapeutic drug concentrations in the brain. Nanoparticle-based drug delivery systems have emerged as a promising strategy to overcome these limitations by enhancing CNS drug penetration and selectively targeting metastatic brain tumor cells while minimizing off-target effects. This review summarizes recent preclinical and clinical developments in nanoparticle-based therapies for BM. It is evident from these studies that NPs can carry with them a range of therapeutics, including chemotherapy, immunotherapy, small molecule inhibitors, gene therapies, radiosensitizers, and modulators of tumor microenvironment to the BM. Moreover, preclinical studies have shown encouraging efficacy in murine models, highlighting the potential of these platforms to improve therapeutic outcomes. However, clinical translation remains limited, with few ongoing trials. To close this translational gap, future work must address clinical challenges such as trial design, regulatory hurdles, and variability in BBB permeability while developing personalized nanoparticle-based therapies tailored to individual tumor characteristics.

## 1. Introduction

Brain metastases (BM) occur when cancer cells spread from their primary site, commonly the lung, breast, or skin, to the brain parenchyma [1,2]. Despite the protective nature of the blood–brain barrier (BBB), BM develop in approximately 10–40% of cancer patients and are more common than primary brain tumors [3,4,5]. A hallmark of BM is the disruption of BBB integrity [6]. Tumor cells can recognize and adhere to vascular wall components, triggering a cascade of events that allows them to breach the BBB, invade the brain tissue, and establish BM [7,8]. Lung cancer is the leading cause of brain metastases, accounting for 20–56% of cases [3,4,9,10]. It has the highest incidence of brain spread among all cancers and is the most frequent cause of BM in men [11]. In women, breast cancer (BC) is the predominant primary source [11], responsible for 5–20% of brain metastases [3,4,9,10]. Melanoma also contributes significantly (7–16%) [3,4,9,10]. The incidence of BM from renal cell carcinoma (RCC) and colorectal cancer (CRC) is also significant and increasing [12].

The prognosis for patients with brain metastases remains poor, with a median overall survival (OS) of just 10–16 months, despite advances in treatment [13,14]. Local therapies, such as stereotactic radiosurgery (SRS), surgical resection, and, to a lesser extent, whole-brain radiation therapy (WBRT), continue to form the backbone of treatment [15,16]. Moreover, systemic therapeutic options are rapidly expanding and remain a focus of ongoing clinical research [16]. However, systemic therapy (ST) uptake into the brain is significantly restricted by several factors, most notably physical barriers such as the BBB and the blood–cerebrospinal fluid (blood–CSF) barrier [17,18]. In addition, ST delivery efficiency is influenced by the substrate’s affinity for specific transport systems located at these interfaces [17,18]. These limitations often result in subtherapeutic drug levels in the brain, creating a major barrier to effective treatment of central nervous system diseases, such as brain metastases [19]. In addition, most ST agents are non-specific and are associated with systemic toxicities, presenting a major biomedical challenge in achieving a therapeutically effective concentration that targets tumor cells without causing substantial harm to the patient [20].

To improve drug delivery across the brain’s physical barriers, reduce off-target toxicities, and enhance the intratumoral concentration of ST in BM, nanoparticles (NP) offer a highly promising approach [19,21,22]. These platforms can be engineered to carry diverse payloads for combination therapies and selectively target metastatic lesions. Moreover, they enable spatiotemporally controlled drug release [22] by responding to specific tumor microenvironment (TME) cues, such as temperature, acidic pH, matrix metalloproteinase (MMP), or reactive oxygen species [23,24,25,26], as well as to externally applied stimuli, including light, X-rays, ultrasound, or magnetic fields [22,27,28].

## 2. Structure of Nanoparticles

**Figure 1:**
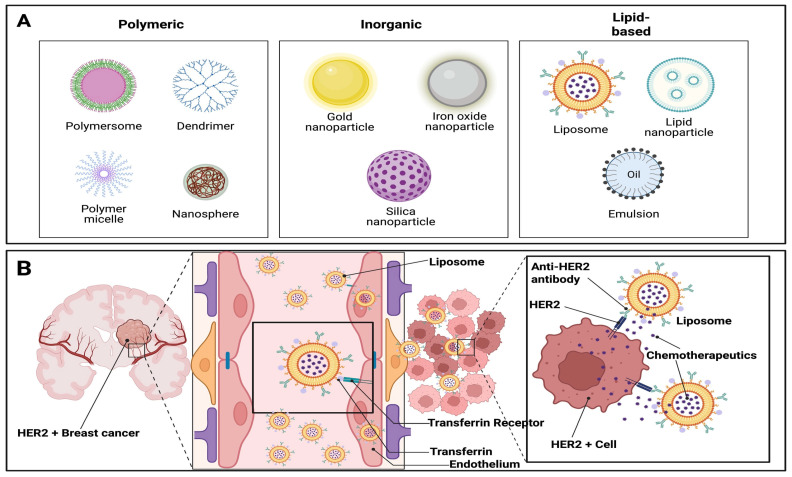
**Classification and Mechanism of Nanoparticles**: (**A**) illustrates three different types of NP: (1) polymeric (e.g., polymersomes, dendrimers), (2) inorganic (e.g., gold, iron oxide), and (3) lipid-based (e.g., liposomes, emulsions), each offering unique properties for drug delivery. (**B**) illustrates the mechanism by which liposomal NPs deliver chemotherapeutics across the BBB to target HER2-positive breast cancer metastases in the brain. Liposomes are engineered to interact with transferrin receptors expressed on the endothelial cells of the BBB, facilitating receptor-mediated transcytosis. After crossing the BBB, the liposomes accumulate in the tumor microenvironment and specifically bind to HER2-expressing cancer cells through anti-HER2 antibodies conjugated to their surface. This targeted interaction allows for localized drug release into the tumor cells, thereby enhancing therapeutic efficacy while minimizing off-target toxicity and preserving healthy tissue.

NPs are broadly categorized into three main types based on their composition and structure (Figure 1A) [29]. (1) Lipid-based NPs (e.g., liposomes, lipid NPs, and emulsions) are typically spherical structures composed of at least one lipid bilayer encasing an internal aqueous compartment [29,30]. These systems offer numerous advantages, including ease of formulation, self-assembly, high biocompatibility, excellent bioavailability, and the capacity to carry large therapeutic payloads [30]. Their physicochemical properties can also be tailored to influence biological behavior, making them the most commonly FDA-approved nanomedicine platforms [31]. (2) Polymeric NPs (e.g., poly(lactic-co-glycolic acid) and poly(methacrylic acid) are synthesized from natural or synthetic polymers, using either monomers or preformed polymers [29]. Their versatility allows for fine-tuned control over characteristics such as size, surface charge, and drug release profiles [29]. These NPs are manufactured using methods like emulsification, nanoprecipitation, ionic gelation, and microfluidics [29]. Polymeric NPs are highly adaptable drug delivery systems capable of encapsulating, entrapping, conjugating, or surface-loading both hydrophilic and hydrophobic agents, including small molecules, proteins, biological macromolecules, and vaccines, making them ideal for combination therapies [29]. (3) Inorganic NP, composed of materials such as gold, iron, or silica, are engineered with precise control over size, shape, and geometry for both drug delivery and imaging applications [29]. Gold NPs (AuNP) are extensively studied and can take on various forms like nanorods, nanostars, and nanoshells. These inorganic systems possess unique physical and chemical properties derived from their base materials, such as optical, magnetic, and photothermal behaviors [29]. For instance, AuNPs exhibit size- and shape-dependent surface electron oscillations, which contribute to their photothermal effects [29]. They are also readily functionalized to enhance targeting and therapeutic capabilities [29]. Iron oxide is another widely studied material for the synthesis of inorganic NPs, and iron oxide-based NPs constitute most FDA-approved inorganic nanomedicines [29]. Other commonly used inorganic NPs include calcium phosphate and mesoporous silica NPs, both of which have been successfully employed in gene and drug delivery applications [29]. The structure, cargo, and mechanism of action (Figure 1B) of each NP used for BM are further detailed in the sections below.

## 3. Factors Affecting the Biodistribution of Nanoparticles

The BBB is a highly selective and semi-permeable barrier that separates the circulating blood from the brain and the extracellular fluid in the CNS [32]. The physicochemical properties of this natural barrier are crucial for designing drugs and carriers that target the treatment of CNS diseases. Certain characteristics of the BBB can influence the ability of NPs to penetrate the CNS. For instance, the outer components of the BBB possess a net negative surface charge, which prevents the uptake of negatively charged compounds [33,34,35]. Moreover, the BBB contains specific transporters that regulate the influx and efflux of substances into and out of the CNS. These transporters are categorized into ATP-binding cassette (ABC) transporters and ATP-binding cassette subfamily B member (ABCB) transporters, which facilitate the entry of large endogenous molecules such as transferrin and insulin. Conversely, P-glycoprotein (P-gp) regulates the efflux of most other substances. Moreover, passive diffusion is limited to low molecular weight compounds (less than 400 Da) that are nonpolar and lipophilic [33,34]. In contrast, water-soluble or polar compounds can only cross the BBB through active transport systems [35,36].

Similarly, the physicochemical properties of NPs, particularly their size, shape, and surface charge, significantly influence their biodistribution and therapeutic efficacy [37]. Smaller-sized NPs tend to accumulate more readily in organs such as the liver, lungs, spleen, and kidneys, and are also more capable of crossing the BBB [38,39]. However, these small NPs are often associated with increased off-target toxicity [40]. To mitigate such adverse effects, NPs should ideally be larger than the pore size of normal blood vessels (typically 6–12 nm) to reduce off-target tissue penetration [41], yet small enough to pass through leaky vessels in the tumor where vessel pore sizes range from 40 to 200 nm and exploit the enhanced permeability and retention (EPR) effect in tumor tissues [39]. Furthermore, NP size influences the clearance of NPs [37]. NPs smaller than 5–6 nm are rapidly cleared through renal filtration, while NPs with a size of 200 nm or larger are predominantly removed by the reticuloendothelial system (RES), which involves macrophages in the liver, spleen, and bone marrow [42,43]. The shape of NPs also plays a pivotal role in their biodistribution and immune evasion [44]. Elongated NPs with a high length-to-width ratio are generally less prone to clearance by the immune system, as they are more likely to evade recognition and uptake by macrophages [45]. Therefore, these rod-shaped or filamentous NPs tend to exhibit longer blood circulation times [46]. When NPs encounter macrophages, the initial contact angle (θ) strongly dictates the rate of internalization. For rod-shaped NPs, alignment parallel to the cell membrane (long axis parallel) leads to slower uptake, whereas perpendicular alignment (θ = 90°) significantly enhances phagocytosis. In contrast, spherical NPs, due to their symmetrical shape, are internalized at a rate that is independent of contact angle (θ) [47,48]. Similarly, the surface charge of NPs, quantified as zeta potential (ξ), affects their stability, biodistribution, and clearance [49]. Positively charged NPs (ξ > +10 mV) tend to induce serum protein aggregation [49], promoting opsonization and rapid clearance and affecting biodistribution, toxicity, and the therapeutic efficacy of NPs [50]. In contrast, negatively charged NPs (ξ < −10 mV) exhibit strong uptake by the RES [49]. Neutrally charged NPs (ξ within ± 10 mV) demonstrate minimal RES interaction and typically exhibit the longest circulation times [49]. Surface charge also influences electrostatic interactions with biological tissues, especially in TME [37]. Positively charged NPs may be attracted to negatively charged cell membranes, while negatively charged NPs may experience repulsion, affecting tissue penetration and cellular uptake. Therefore, the neutral surface charge can be beneficial for reducing non-specific interactions and avoiding premature clearance [51]. Moreover, hepatic clearance is also strongly influenced by surface charge [52]. NPs with high positive (>+10 mV) or negative (<−10 mV) zeta potentials are efficiently sequestered by Kupffer cells in the liver [52]. Therefore, it is important to consider these factors while designing NPs for BM.

## 4. Overview of Pre-Clinical Trials Using Nanoparticles for Brain Metastases

### 4.1. Overview of Nanoparticles Carrying Chemotherapeutic Agents

Although multiple chemotherapeutics have been explored for treating BM, their effectiveness remains limited due to several challenges, including poor permeability of most drugs across the BBB, which restricts adequate drug accumulation within metastatic brain tissue, and off-target toxicities [17,18,20]. Consequently, NPs have been engineered to facilitate drug delivery across this physiological barrier, a critical step toward improving therapeutic outcomes in the BM [19,21,22]. For instance, Zhang et al. [26] designed lexiscan-encapsulated, AMD3100-conjugated, shrinkable NPs (LANP), which were loaded with doxorubicin (Dox) to target breast cancer brain metastases (BCBM). The concentration of LANP in BCBM was over 2.5 times higher than in peripheral organs. Treatment with Dox-LANP significantly prolonged the survival of mice bearing BCBM (*p* < 0.05). Histological analysis with H&E and Ki67 staining demonstrated that, compared to the control PBS group, tumors in the Dox-LANP-treated group exhibited significantly fewer lesions, which were also smaller. Additionally, TUNEL staining revealed significant apoptosis in the tumors of the Dox-LANP group, whereas no such effect was observed in the control group. Moreover, treatment with free Dox significantly reduced mouse body weight. Histological analysis revealed that this side effect was due to substantial myocardial injury, a well-known dose-limiting toxicity associated with in vivo Dox administration. In contrast, doxorubicin-loaded LANP (Dox-LANP) did not induce detectable myocardial toxicity or cause damage to normal organs. Li et al. [53] engineered PMAA-PS 80-g-St terpolymers (PPT) and found that these NPs adsorb apolipoprotein-E (Apo-E) from plasma, which facilitates their uptake by the BBB and BM via LDL receptors found on the cell surfaces. In vivo, a mouse model with intracranial BCBM was used to study the pharmacokinetics and efficacy of PPT [53]. The ability of the PTT to cross the intact brain microvasculature in healthy mice was confirmed through in vivo MRI and ex vivo confocal microscopy [53]. Dox-loaded PTT rapidly accumulated within metastatic lesions and released Dox locally, confirmed by histological and fluorescence microscopy analyses [53]. In contrast, no Dox was detected in tissues treated with free Dox [53]. Moreover, significant apoptosis was observed in both large metastases and micro-metastases within 24 h of NP treatment, while minimal apoptosis was seen in normal brain tissue, demonstrating tumor-selective cytotoxicity [53]. In comparison, only sparse apoptotic cells were present in brains treated with free Dox [53]. In addition, treatment with Dox-loaded PPT significantly reduced brain tumor burden in NRG-SCID mice compared to free Dox [53]. Both in vivo and ex vivo imaging showed minimal NP uptake by the liver and even lower levels in the spleen [53]. The relatively higher fluorescence intensity observed in the gallbladder and kidneys at 2 h post-injection suggests that the NPs are likely eliminated via both biliary and renal pathways [53]. Zhang et al. [54] modified the previously reported PPT developed by Li et al. [48] by conjugating it with iRGD. The iRGD moiety binds to αv integrins expressed on the surface of tumor cells, thereby enhancing NP uptake by BM cells [54]. Simultaneously, Apo-E facilitates the internalization of NPs by brain endothelial cells of the BBB and by tumor-associated macrophages (TAM) within the TME [54]. The iRGD-PPT system was co-loaded with Dox and mitomycin C (MMC) to dual-target triple-negative breast cancer (TNBC) cells and TAM [54]. This formulation exhibited enhanced BBB penetration, resulting in higher BM drug concentrations [54]. In vivo, BM-bearing mice treated with iRGD-PPT showed reduced tumor progression and prolonged survival [54].

Geretti et al. [55] investigated a strategy to improve the efficacy of HER2-targeted PEGylated liposomal doxorubicin (PGL/MM-302) in HER2-positive breast cancer by combining it with a tumor-priming dose of cyclophosphamide (CPA). MM-302 is an NP drug formulation engineered to selectively deliver doxorubicin to HER2-overexpressing tumor cells while minimizing exposure to healthy tissues [55]. The study demonstrated that pretreatment with CPA significantly enhanced MM-302 accumulation within tumors by approximately 2- to 3-fold, without increasing drug levels in off-target tissues like the heart or skin [55]. The results indicated that CPA had no observable effect on HER2-tPLD pharmacokinetics, thus confirming that the measured changes in delivery are associated with modifications in the TME rather than with a delay in clearance of HER2-tPLD [55]. Mechanistically, this tumor priming effect was shown to rely on CPA-induced apoptosis in tumor cells, which led to a reduction in cell density, decreased interstitial fluid pressure, and increased vascular perfusion, particularly in smaller tumor blood vessels [55]. This remodeling of the TME improved the extravasation and penetration of MM-302 [55]. Notably, these effects were time-sensitive, with the greatest benefit observed when CPA was administered 2–5 days before injection of HER2-tPLD (MM-302) [55]. The percentage of doxorubicin-positive nuclei increased 12-fold for the CPA-treated group [55]. Functionally, this combination therapy resulted in significantly greater nuclear doxorubicin delivery, increased DNA damage and apoptosis (as shown by γ-H2AX and cleaved caspase-3 staining), and enhanced tumor growth inhibition compared to either agent alone [55]. Notably, this approach avoided cardiotoxicity typically associated with free doxorubicin [55]. In summary, the study supports CPA-induced tumor priming as a viable and translatable method to improve the therapeutic index of nanoparticle-based chemotherapies such as MM-302 in HER2-positive breast cancer [55].

Sambade et al. [56] developed and tested acid-labile C2-docetaxel-loaded PRINT^®^ NP (PRINT-C2-DTX) for the treatment of BM from non-small-cell lung cancer (NSCLC). These NPs were designed using the Particle Replication In Nonwetting Templates (PRINT) technology to create PLGA-based cylindrical particles loaded with either standard molecule docetaxel (SM DTX) or the C2-docetaxel (C2-DTX) prodrug [56]. The C2-DTX is an acid-sensitive prodrug that selectively converts to active DTX in the acidic TME, enhancing tumor specificity and minimizing systemic toxicity [56]. PRINT-C2-DTX extended median survival by 35% while exhibiting lower toxicity compared to the other treatment groups [56]. In a murine A549-luc NSCLC intracranial tumor model, PRINT-C2-DTX NP achieved over sevenfold higher tumor drug concentrations compared to free SM DTX [56]. Notably, the conversion of C2-DTX to active DTX was over three times higher in tumor tissue than in non-tumor tissues, demonstrating successful tumor-selective activation [56]. Treatment with PRINT-C2-Dox significantly improved median survival (90 days) compared to SM-docetaxel (66.5 days), PRINT-docetaxel (58.5 days), and vehicle control (61 days) [56]. While all treatments reduced tumor burden, only PRINT-C2-DTX extended survival, which was attributed to reduced toxicity and prolonged, controlled drug release [56]. Weight monitoring showed that mice receiving PRINT-C2-DTX maintained stable body weight, unlike other groups [56]. In contrast, SM DTX and standard PRINT-DTX were associated with weight loss and systemic toxicity [56]. Thus, this formulation demonstrates a promising strategy to enhance therapeutic efficacy while reducing the adverse effects of chemotherapy in BM treatment [56].

Wang et al. [57] designed a redox-responsive, targeted nanocarrier system (T7-DSNP/9291) for the co-delivery of Osimertinib (AZD9291) and Dox to treat BM from NSCLC. These NPs are based on a self-assembling Dox prodrug backbone (Dox-SS-C18), which enables drug release in response to high intracellular glutathione (GSH) levels, a common feature of tumor cells [57]. The NP is further functionalized with T7 peptide, which targets the transferrin receptor, enhancing BBB penetration of AZD9291 and Dox via receptor-mediated transcytosis. Following intravenous administration, the T7-modified NPs remain stable in circulation, preventing premature drug release [57]. Upon reaching the brain and crossing the BBB, T7-DSNP/9291 are internalized by tumor cells, where the high GSH environment triggers disulfide bond cleavage, releasing AZD9291 and Dox directly within tumor cells [57]. AZD9291 inhibits EGFR signaling, including T790M mutations, while Dox induces DNA damage, leading to enhanced tumor cell apoptosis [57]. In vitro, T7-DSNP/9291 identified superior uptake and BBB penetration compared to untargeted controls and exhibited strong cytotoxicity against NSCLC (PC-9) cells [57]. T7-DSNP/9291 exhibited minimal toxicity toward mouse BCEC cells, likely due to the low intracellular GSH levels in these cells, which are insufficient to cleave the disulfide bonds and trigger the release of AZD9291 and Dox [57]. In contrast, free AZD9291 and Dox markedly suppressed the growth of BCEC cells when administered at the same concentration [57]. In vivo, intracranial PC9 tumor mouse models revealed significantly reduced tumor burden and prolonged survival [57]. Mice treated with T7-DSNP/9291 showed a median survival of 35 days compared to 21 days for saline and 24–28 days for other treatment groups [57]. Notably, no major off-target toxicity was observed [57]. These results underscore T7-DSNP/9291 as a promising brain-targeted therapeutic strategy, capable of delivering synergistic drug combinations directly to BM, enhancing efficacy while minimizing systemic toxicity. Similarly, many other pre-clinical trials [58,59,60,61,62,63,64,65,66,67,68,69] have shown efficacy in vivo, and their details are given in Table 1.

### 4.2. Overview of Nanoparticles Carrying Immunotherapeutic Agents

Therapeutic monoclonal antibodies (mAbs) have transformed cancer treatment, but their efficacy against CNS metastases remains limited due to poor CNS penetration, typically achieving only ~0.1% of plasma concentrations [70]. Bypassing the BBB via intrathecal or intraventricular administration can enhance mAb delivery and has demonstrated some success against CNS metastases [70,71,72,73]. However, these methods are invasive and are constrained by the rapid efflux of mAbs from the CNS within hours [74]. Therefore, NP-based delivery systems are emerging as promising strategies enabling efficient and sustained mAb delivery to the CNS while maintaining systemic therapeutic efficacy.

Trastuzumab (TRZ), a monoclonal antibody used to treat HER2-positive breast cancer, has very limited ability to cross the BBB due to its large molecular size (~150 kDa) [75]. Sevieri et al. [76] developed ferritin-based nanoconjugates (H-TZ) to enhance the delivery of TRZ, an anti-HER2 monoclonal antibody, to HER2+ BCBM. The nanocarrier was created by covalently attaching TRZ to human ferritin (HFn) NPs using a PEG-based crosslinker [76]. These HFn NPs naturally target the transferrin receptor 1 (TfR1), which is highly expressed on both the BBB and many tumor cells, enabling H-TZ to cross the BBB and specifically bind to HER2+ tumor cells [76]. Once systemically administered, H-TZ accumulates efficiently in BM, enhancing TRZ distribution in the brain [76]. Combined with docetaxel, H-TZ induced significant tumor suppression in vivo, outperforming free TZ with docetaxel in reducing tumor burden and promoting a protective immune response [76]. This study examined the effect of H-TZ+DTX treatment on Iba1, a microglia/macrophage marker, revealing increased Iba1-positive areas in the peri-tumoral regions of treated mice [76]. Specifically, the H-TZ nanoconjugates enhanced macrophage activation (via Iba1 positivity) and modulated neuroinflammation by increasing protective cytokines (IL-8, IL-10) and suppressing proinflammatory cytokines (TNFα, IL-2, IFN-γ) [76]. Importantly, H-TZ delivery significantly reduced off-target accumulation of TRZ in the heart, avoiding the cardiotoxic mitochondrial damage typically associated with standard therapy [76]. No liver or kidney toxicity or other systemic side effects were observed [76]. He et al. [77] developed a terpolymer-based NP (TRZ–TPN) for the targeted delivery of TRZ to BM in HER2-positive breast cancer, achieving high encapsulation efficiency while preserving antibody bioactivity. In vivo studies demonstrated that TRZ–TPN delivered approximately 50-fold higher TRZ concentrations to BM compared to free TRZ [77]. Treatment with TRZ–TPN resulted in a 4-fold increase in tumor cell apoptosis, a 43-fold reduction in tumor growth, and a >1.3-fold improvement in median survival, all without inducing observable organ toxicity [77]. Similarly, Wyatt and Davis et al. [78] designed mucic acid polymer-based NPs to carry Herceptin (anti-HER2 TRZ) and Camptothecin (topoisomerase I inhibitor) and target HER2+ BCBM. In vivo, combined Camptothecin (CPT)/Herceptin-loaded NPs demonstrated the most significant tumor inhibition and a durable therapeutic response, outperforming NPs loaded with either CPT or Herceptin alone [77]. The combination of free CPT and Herceptin showed inferior efficacy compared to the dual-loaded NP formulation, highlighting the benefits of co-delivery via nanocarriers [77].

Rituximab (RTX) was the first anti-cancer antibody approved by the U.S. Food and Drug Administration for the treatment of non-Hodgkin lymphoma (NHL). It targets CD20-positive lymphoma cells and induces cell death via complement-dependent cytotoxicity (CDC), antibody-dependent cell-mediated cytotoxicity (ADCC), and apoptosis [79]. While RTX has shown significant therapeutic benefits in systemic NHL, its effectiveness in treating primary and relapsed CNS lymphoma has been limited when administered intravenously, likely due to minimal penetration of RTX into the CNS [70]. To improve CNS penetration, Wen et al. [74] designed CXCL13-conjugated RTX NPs (n-RTX^CXCL13). CNS accumulation of RTX increased approximately 10-fold with n-RTX^CXCL13 compared to native RTX [74]. Moreover, in vivo n-RTX^CXCL13 significantly reduced brain lymphoma burden and achieved complete clearance of CNS lymphoma in BLT humanized mouse models [74]. Survival was significantly improved in both NSG and BLT mouse models following treatment with n-RTX^CXCL13 formulations [74].

STAT3 is aberrantly activated in both tumor cells and astrocytes, where it drives the production and secretion of pro-tumorigenic signaling molecules, contributing to the formation of an immunosuppressive metastatic microenvironment [80,81]. In metastatic tumor cells, STAT3 activation promotes chemoresistance and stimulates the release of growth factors that, in turn, activate STAT3 in astrocytes [82,83]. These STAT3-activated astrocytes secrete macrophage migration inhibitory factor (MIF) and pro-inflammatory cytokines such as transforming growth factor-β (TGF-β), which act on TAM, promoting their M2-like immunosuppressive phenotype and facilitating immune cell exhaustion [80,84]. Silibinin (SIL), a STAT3 inhibitor, disrupts these signaling axes in the brain microenvironment and has demonstrated efficacy in improving outcomes in BCBM [85]. Zhao et al. [86] developed CSKC peptide-functionalized NPs (SIL@T NP) co-loaded with SIL and oxaliplatin (OXA) to target breast cancer brain metastases. SIL, a STAT3 inhibitor, reverses the immunosuppressive TME, while OXA, a platinum-based chemotherapeutic, induces immunogenic cell death (ICD) [86]. The CSKC cyclic peptide, an analog of the IGF-1R ligand, was conjugated to the distal end of PEG to serve as a targeting moiety, enhancing BBB penetration and selective accumulation in BM due to the high expression of IGF-1R on BBB endothelial cells and BM [86]. In the acidic environment of endosomes or lysosomes, protonation of the lysine side chain triggers micelle dissociation and efficient intracellular release of SIL [86]. In vitro, SIL@T NPs effectively inhibited STAT3 activation and induced ICD in tumor cells [86]. In vivo, treatment with SIL@T NPs suppressed metastatic tumor growth and significantly prolonged median survival in a mouse model of BM [86]. Further details on these NPs are given in Table 2.

### 4.3. Overview of Nanoparticles Carrying Small-Molecule Inhibitors

Afatinib (Afa) was approved by the FDA in 2013 as a first-line treatment for patients with EGFR-specific mutations, particularly exon 19 deletions or exon 21 (L858R) substitutions [87]. However, its clinical use is associated with significant side effects, including grade 3–4 diarrhea, rash or acne, and paronychia [88]. The dose-limiting gastrointestinal toxicities, along with the adverse effects resulting from irreversible inhibition of the tyrosine kinase domain of EGFR, often restrict the broader clinical application of Afa at higher doses [89]. To manage these toxicities, dose reductions are commonly required, but lowering the dose can lead to subtherapeutic drug concentrations in the cerebrospinal fluid (CSF), ultimately contributing to treatment failure against BM [90]. Lo et al. [89] developed two lipid-polymeric NPs (LPNs)—Afa/LPN-FD7 and Afa/LPN-CCD—functionalized with tight junction-modulating peptides (FD7 and CCD) to enhance the delivery of Afa across the BBB for the treatment of EGFR-positive NSCLC BM and reduce its adverse effects. In vitro, these LPNs successfully crossed a BBB model using bEnd.3 cells and significantly enhanced afatinib’s cytotoxic effect on PC9 cells [89]. The peptides FD7 and CCD disrupted tight junction (TJ) proteins, such as claudin-5 and ZO-1, resulting in a decrease in transendothelial electrical resistance (TEER) and an increase in FITC-dextran permeability, suggesting that these NPs primarily traverse the BBB via a paracellular route by loosening TJ interactions without altering the overall expression of TJ proteins [89]. The LPNs were also shown to undergo partial transcellular transport via clathrin- and caveolae-mediated transcytosis, indicating a dual mechanism for BBB penetration [89]. The LPNs were not tested in vivo to study the survival benefits in animal models.

Ligand-functionalized NPs designed to cross the BBB typically achieve less than 1% of the injected dose accumulating in brain tissue after systemic administration [91,92,93]. It has been hypothesized that abluminal low-density lipoprotein receptor-related protein 1 (LRP1), which functions as a clearance receptor for amyloid-beta (Aβ) [94,95,96] and tissue plasminogen activator (tPA) [97], may bind to NPs after they cross the BBB, promoting their clearance from the brain to the bloodstream via clearance transcytosis [93]. This mechanism likely contributes to the low accumulation of NPs in the brain parenchyma [93]. To overcome this, Khan et al. [93] developed a fusion peptide K-s-A, integrating the HER2-targeting sequence KAAYSL (K), an MMP1-sensitive linker VPMS-MRGG (s), and LRP1-targeting angiopep-2 (A) to design poly(lactic-co-glycolic acid)-poly(ε-carbobenzoxy-l-lysine) (PLGA-PLL) NPs (NP-K-s-A). These engineered NPs first cross the BBB through interaction with luminal LRP1, and upon encountering the matrix metalloproteinase 1 (MMP1)-rich microenvironment of BCBM, the MMP1-sensitive linker cleaves, releasing angiopep-2 and thereby evading abluminal LRP1-mediated clearance [93]. The exposed KAAYSL peptide then enables targeted binding to HER2-expressing BCBM, enhancing NP accumulation in brain lesions [93]. In vivo studies demonstrated that NP-K-s-A achieved fivefold higher brain accumulation in BCBM-bearing mice than the MMP1-insensitive control NPs (NP-K-i-A), highlighting the importance of evading abluminal clearance [93]. Furthermore, treatment with Dox and lapatinib (Lap) co-loaded in NP-K-s-A significantly prolonged the survival of BCBM-bearing mice compared to controls, confirming the therapeutic advantage of this design [93].

Yin et al. [98] developed a dual-targeting liposomal codelivery system (T12/P-Lipo) modified with a transferrin receptor TfR-binding peptide (T12) to enhance BBB penetration and an anti-PD-L1 nanobody to target TAM and BM of EGFR T790M-mutated NSCLC. The liposomes were loaded with simvastatin (SV) and gefitinib (Gef) [98]. SV-based treatment to repolarize the TAM from the M2 to M1 phenotype [99] and re-sensitize the T790M mutated cells to Gef therapy [98]. The NPs efficiently penetrated the BBB and achieved high accumulation in brain tumors by targeting both TAM and BM via TfR and PD-L1 receptors [98]. TAM plays an essential role in regulating the TME [98]. In vitro, T12/P-Lipo induced repolarization of TAM from the pro-tumorigenic M2 to anti-tumorigenic M1 phenotype, elevated intracellular reactive oxygen species (ROS), and inhibited the EGFR/Akt/Erk signaling pathway, thereby reversing drug resistance to Gef [98]. In vivo studies using an intracranial H1975 xenograft model demonstrated that T12/P-Lipo significantly prolonged survival, enhanced tumor apoptosis (TUNEL staining), and reduced tumor proliferation (Ki-67 staining) with minimal toxicity observed in major organs [98].

Zhao et al. [100] developed a biomimetic, BBB-permeable NP system (T12-BSA NP) for co-delivery of regorafenib (Rego) and disulfiram/copper (DSF/Cu) to overcome resistance to osimertinib in NSCLC BM. Rego acts as an antiangiogenic drug by inhibiting the VEGF/VEGFR pathway, while disulfiram (DSF) targets FROUNT, a protein highly expressed in M2-like macrophages [100]. These NPs are modified with T12 peptide, allowing them to selectively target tumor cells and TAM via Secreted Protein Acidic and Rich in Cysteine (SPARC) and TfR receptors, enabling dual targeting [100]. Mechanistically, the therapeutic effect relied on repolarizing CD206hi TGF-β1^+^ M2-type TAM to antitumoral M1-type macrophages by inhibiting FROUNT and reducing proangiogenic vascular endothelial growth factor (VEGF) secretion, thus remodeling TME [100]. In vivo, T12-BSA NPs demonstrated significantly enhanced accumulation in the subcutaneous xenograft and BM model, with 2-fold higher delivery efficiency than unmodified NPs [100]. Treatment with T12-BSA NPs resulted in substantial tumor growth inhibition and extended survival in osimertinib-resistant NSCLC BM models, increasing median survival to 36 days without inducing systemic toxicity [100].

Wan et al. [101] developed lapatinib-loaded human serum albumin (HSA) NPs (LHNP) using a modified NP albumin-bound (Nab) technology to enhance drug delivery to prevent and treat TNBC BM. These LHNPs consist of a core–shell structure where Lap is encapsulated within a matrix of HSA and phosphatidylcholine, forming stable nanoparticles capable of evading efflux mechanisms at the BBB [101]. LHNP exploits the enhanced permeability and retention (EPR) effect and albumin-mediated transport (via gp60 and SPARC interactions) to accumulate in tumor tissues [101]. Compared to free Lap, LHNP significantly enhanced drug delivery to the brain, overcoming the BBB and P-glycoprotein-mediated efflux [101]. Once delivered, Lap inhibits EGFR and HER2 signaling pathways, which are often overexpressed in TNBC and contribute to metastatic progression [101]. In vitro, LHNP could maintain the integrated structure of NPs in the bloodstream for hours and inhibit the adhesion, migration, and invasion of 4T1 breast cancer cells more effectively than free drug [101]. In vivo, LHNP achieved 5.4-fold higher accumulation in BM compared to oral Lap and extended median survival of BM-bearing mice from 19.1 days (control group) to 36.4 days with high-dose LHNP [101]. Moreover, LHNP significantly downregulated key metastasis-related proteins, including MMP-2, uPA, OPN, and CXCR4. LHNP demonstrated a favorable safety profile, with no significant toxicity or histopathological abnormalities observed in major organs [101]. Further details on these NPs are given in Table 3.

### 4.4. Overview of Nanoparticles Carrying Therapeutic Genes

The development of antisense oligonucleotides (ASOs) for inhibiting protein synthesis in the early 1980s marked the beginning of RNA-based therapeutics [102]. In the 2000s, the discovery of RNA interference (RNAi) and the application of small interfering RNAs (siRNAs) for human gene silencing sparked significant interest and investment in the field [102]. Today, RNA-based therapeutics encompass a wide range of modalities, including ASOs, siRNAs, microRNAs (miRNAs), long noncoding RNAs (lncRNAs), aptamers, ribozymes, messenger RNA (mRNA), and CRISPR/Cas9 systems [103]. Despite their promise, RNA therapeutics face several major challenges, particularly for neurological diseases, including BM [103]. These include rapid enzymatic degradation, limited permeability across the BBB, unintended on- and off-target effects, inefficient cellular uptake, and endosomal escape [103]. Overcoming these hurdles is essential for successful clinical translation. Various delivery strategies, particularly NP-based systems, have been explored to address these limitations [103,104]. NPs can protect RNA molecules from degradation, enhance BBB penetration, and improve cellular uptake and release. In recent years, preclinical studies have investigated NP-encapsulated RNA-based therapeutics for the treatment of BM, showing promise in improving targeting, stability, and therapeutic efficacy [103,104]. For example, Zhao et al. [105] developed T-M/siRNA micelles co-loaded with siRNA targeting protocadherin 7 (PCDH7) and the chemotherapeutic agent paclitaxel (PTX) [105]. The siRNA component was designed to suppress PCDH7, a key protein involved in GJs formation, thereby disrupting intercellular communication between astrocytes and BM cells [105]. PTX served as the cytotoxic agent to exert direct anti-tumor effects [105]. The disulfide bonds in T-M/siRNA micelles are cleaved under high intracellular GSH conditions in BM, triggering the release of PTX and promoting micelle disintegration [105]. This process reduces the micelle’s charge density, thereby facilitating the accelerated release of siRNA targeting PCDH7 [105]. In vitro studies demonstrated that T-M/siRNA micelles exhibited efficient cellular uptake, significantly enhanced cytotoxicity against MDA-MB-231/Luc cells, and effectively reduced the expression of PCDH 7 and GJs formation [105]. In vivo bioluminescence imaging confirmed that the micelles could successfully cross the BBB and release therapeutic payloads at the tumor site [105]. Notably, mice treated with T-M/siRNA micelles showed a prolonged median survival compared to control groups [105].

MicroRNA-10b is a well-established oncogenic microRNA implicated in promoting metastatic dissemination [106] and supporting the viability of metastatic cells [107]. It has been validated as a biomarker correlated with various clinical parameters, including disease stage, presence of metastases, relapse-free survival, overall survival, invasiveness, and time to recurrence [108]. In a notable study, Yoo et al. [109] developed modified magnetic NPs (MNs) by conjugating them with the heterobifunctional linker N-succinimidyl 3-[2-pyridyldithio]-propionate (SPDP) to load anti-miR-10b antagomirs, resulting in a formulation termed MN-anti-miR-10b for targeted delivery to BM. In vivo fluorescence optical imaging (FLI) confirmed that MN-anti-miR-10b successfully crossed the BBB and accumulated within BM lesions [109]. The therapeutic efficacy was monitored using bioluminescence imaging (BLI), which revealed a significant reduction in metastatic burden as early as after the first treatment [109]. After three weeks of therapy, the MN-anti-miR-10 b-treated group exhibited a markedly lower metastatic load compared to the control group treated with MNs. Notably, the metastatic burden after treatment was significantly reduced compared to baseline levels, indicating that the viable tumor mass regressed during therapy [109].

Lysophosphatidylcholine acyltransferase 1 (LPCAT1) is a cytosolic enzyme responsible for converting lysophosphatidylcholine to phosphatidylcholine and is found to be highly overexpressed in various cancers [110,111,112,113]. LPCAT1 has been shown to drive brain metastasis in lung cancer by activating the PI3K/AKT/MYC signaling pathway, making it a potential therapeutic target for the BM [110]. Notably, silencing LPCAT1 expression significantly inhibited tumor cell proliferation in vitro and suppressed brain metastatic progression in vivo [110]. To target LPCAT1 in BM, Jiang et al. [114] engineered exosomes (Exo^scFv^) by incorporating an anti-EGFR single-chain variable fragment (scFv) fused to the exosomal membrane protein Lamp2b, which is naturally abundant on exosomal surfaces. In vitro, the targeted uptake of Exo^scFv^ was evaluated using the EGFR-positive PC9 lung cancer cell line, demonstrating efficient internalization [114]. In vivo studies confirmed that EGFR-scFv modification significantly enhanced tumor targeting and prolonged the retention of Exo^scFv^ within the TME [114]. To achieve gene silencing, LPCAT1-targeting siRNAs were loaded into Exo^scFv^ by electroporation, resulting in Exo^scFv/siLPCAT1^ [114]. In vitro, treatment with Exo^scFv/siLPCAT1^ led to a substantial reduction in LPCAT1 expression in PC9 cells and significantly inhibited cell proliferation compared to controls [114]. In vivo imaging confirmed these findings, showing a notably reduced tumor burden in mice following treatment with Exo^scFv/siLPCAT1^ [114].

Ngamcherdtrakul et al. [115] developed a multifunctional nanoparticle formulation, T-siHER2-NP(DTX), designed to co-deliver HER2-targeted siRNA, docetaxel (DTX), and TRZ to HER2-positive BC and BCBM. In vitro, treatment with T-siHER2-NP(DTX) induced apoptotic cell death in the majority of HER2-positive BT474 cells [115]. To enhance BBB penetration in vivo, microbubble-assisted focused ultrasound (MB-FUS) was employed [115]. Mice treated with the combination of MB-FUS and T-siHER2-NP(DTX) exhibited marked inhibition of intracranial BTGFL1 tumor growth throughout the treatment period [115]. The T-siHER2-NP(DTX) approach significantly improved survival compared to either treatment alone, with median survival times of 80 days for the combination group versus 64.5 days for MB-FUS alone and 54 days for T-siHER2-NP(DTX) alone [115].

Melittin, a potent antitumor peptide, has shown promise in cancer therapy, but its clinical application has been limited by significant non-specific cytotoxicity [116,117]. This cytolytic activity can be neutralized by flanking melittin with short peptide sequences [118]. Leveraging this strategy, Zhou et al. [118] engineered an artificial gene, proMel, to express a secretory promelittin protein within tumor cells. While promelittin itself exhibits minimal toxicity, it is selectively cleaved by MMP-2, overexpressed in the tumor microenvironment, to release cytolytic melittin [118]. Moreover, they designed AMD3100-conjugated NPs (AP30NP) to carry proMel to tumor-specific cells [118]. In vivo bioluminescence imaging of luciferase activity demonstrated that treatment with proMel NP significantly inhibited tumor progression [118]. Histological analysis using H&E and TUNEL staining confirmed that proMel NP reduced tumor malignancy and markedly increased apoptotic cell death within the tumor tissue [118]. Importantly, H&E staining also revealed that NP treatment did not cause noticeable damage to the surrounding normal brain tissue [118]. Further details on these NPs are given in Table 4.

### 4.5. Overview of Nanoparticles Carrying Radiotherapeutic/Radiosensitizer

In vitro, combining the AGuIX NP with 2 Gy of radiation resulted in a 52% greater therapeutic effect compared to radiation alone, accompanied by a significant increase in double-strand DNA breaks (DSBs), despite the NPs remaining localized outside the nucleus [119]. In vivo MRI confirmed the accumulation of NPs in B16F10 brain metastases, with uptake progressively increasing for over 3.5 h, as validated by two-photon confocal imaging [119]. The MRI signal persisted for 24 h post-injection, enabling therapeutic irradiation to be conducted over at least two consecutive days [119]. In a treatment mouse model, administration of 7 Gy alone extended the lifespan of mice with multiple brain melanoma metastases by 8.3%, while the combined NP and radiation treatment resulted in a 25% increase in survival, representing a threefold improvement in therapeutic efficacy [119].

Iodine NP (INP)-enhanced radiotherapy for BM was investigated using a murine intracranial model of TNBC. Hainfeld et al. [120] developed PEGylated 20 nm iodine NPs capable of selectively accumulating at the tumor periphery following intravenous injection. In athymic nude mice bearing intracranial MDA-MB-231 tumors, microCT imaging demonstrated preferential INP uptake with a mean iodine concentration of approximately 2.9% by weight at the tumor rim, corresponding to a calculated dose enhancement factor (DEF) of ~5.5 (with peaks up to 8.0) [120]. Treatment with a single 15 Gy dose of X-ray irradiation alone modestly extended survival; however, INP preloading followed by irradiation significantly improved therapeutic outcomes, extending median survival from 61 to 85 days and resulting in long-term remission, with 40% of mice surviving 150 days and 30% surviving > 280 days [120]. No overt systemic toxicity was observed [120]. The enhanced therapeutic effect was attributed to passive tumor targeting via the enhanced permeability and retention (EPR) effect, without active targeting ligands [120]. These findings support INP-mediated radiosensitization as a promising strategy to improve radiotherapy efficacy against brain metastases [120].

Chen et al. [121] identified leucine-rich repeat-containing protein 31 (LRRC31) as a potent radiosensitizing gene in BCBM through a genome-wide CRISPR screen. Overexpression of LRRC31 inhibited nonhomologous end-joining (NHEJ) DNA repair by disrupting the recruitment and activation of DNA-dependent protein kinases via direct interaction with Ku70/Ku80 and ATR [121]. Systemic delivery of LRRC31 gene therapy via autocatalytic brain tumor-targeted NPs sensitized intracranial breast cancer tumors to radiation, significantly prolonging survival in mouse models [121]. Further details on these NPs are given in Table 5.

### 4.6. Overview of Nanoparticles Carrying Tumor Microenvironment Modulators

BM cells can exploit the brain’s microenvironment to enhance their survival and proliferation [122]. Astrocytes play a crucial role in maintaining homeostasis under normal conditions but become reactively activated during pathological states to protect neurons from pathological stresses [123]. Interestingly, BM cells can hijack these neuroprotective mechanisms for their benefit [124]. Activated astrocytes secrete various cytokines, including IL-6, IL-1β, and TGF-β, which promote the proliferation of metastatic tumor cells [125]. Beyond paracrine signaling, tumor cells and astrocytes also engage in direct intercellular communication via gap junctions (GJs) [126]. Connexin 43 (Cx43) is the predominant connexin in the brain and has been shown to upregulate in BM and peritumoral astrocytes and mediate selective GJs formation between astrocytes and BM cells, facilitated by protocadherin 7 [127]. This interaction triggers intercellular communication that leads to the upregulation of survival-related genes such as GSTA5, BCL2L1, and TWIST1 in tumor cells, enhancing their resistance to cell death [127]. Moreover, these GJs enable the transfer of cGAMP from tumor cells to astrocytes, inducing the secretion of inflammatory cytokines (IFN-α and TNF-α) that further fuel tumor growth and chemoresistance [128]. Astrocytes also assist tumor cell survival by sequestering excess intracellular Ca^2+^, thereby preventing chemotherapy-induced apoptosis [124]. Given the role of GJ-mediated communication in BM, therapeutically targeting these GJs presents a promising strategy to hinder metastatic progression and improve treatment outcomes. But targeting GJs between astrocytes and BM cells specifically could be challenging due to insufficient drug delivery to the brain [127] and non-specificity. To overcome these challenges and target GJs between BM and astrocytes, Cheng et al. [127] designed LAsomes (Las), combining the cell membranes (CM) of reactive astrocytes (RA) and BM cells (LLC-BrM) with liposomes. LA, being biomimetic, exhibits the potential of homotypic recognition of the CM of astrocytes and BM cells [127]. In vivo and ex vivo imaging revealed significantly higher accumulation of Las in BM compared to conventional liposomes, indicating that Las are capable of passively crossing the BBB [127]. Moreover, under confocal microscopy, Las were found to be distributed to BM cells and astrocytes [127]. Las were then loaded with carbenoxolone (CBX) [127], a semisynthetic derivative of a natural triterpene compound that has been identified as a broad-spectrum inhibitor of connexin channels [129]. In vitro, in the co-culture of astrocytes and LLC-BrM, CBX-Las blocked the transfer of Ca^2+^ and cGAMP, which promote BM proliferation and chemoresistance, and downregulated the expression of Twist 1 and Bcl-xL survival genes in LLC-BrM cells [127]. Moreover, CBX-Las increased the cytotoxicity of docetaxel (DTX)-loaded human serum albumin NP (DNP), dramatically decreasing the viability of LLC-BrM cells in the co-culture compared to the control DNP group [127]. In vivo, DNP in combination with CBX-Las suppressed the rapid progression of BM and enhanced the survival in mice compared to the control group (free CBX+DNP) [127]. Similarly, Zhao et al. [130] developed biomimetic NP (R&B/NP) by coating a drug-loaded core with an erythrocyte-BM hybrid membrane to restore plasmin-mediated attacks against BM. This design resists homotypic aggregation and enables selective binding and penetration of the inflammatory BBB for targeted drug delivery [130]. These NPs, composed of dexamethasone (Dex) and embelin (Emb)-loaded cores coated with hybrid membranes from erythrocytes and MDA-MB-231Br breast cancer cells, demonstrated improved biocompatibility, immune evasion, and selective penetration of the inflammatory BBB [130]. Upon crossing the BBB, R&B/NP released Dex and Emb to inhibit tumor serpin B2 and neuroserpin, restoring local plasmin production [130]. This led to cleavage of L1CAM, limiting tumor spread along vessels, and generation of soluble factor-related apoptosis ligands (sFasL), inducing tumor apoptosis [130]. In vivo, the combined Dex@R&B/NP and Emb@R&B/NP therapy significantly reduced the intracranial metastatic nodule development and extended median survival in mice (46.5 vs. 26 days; *p* < 0.001), without detectable toxicity [130]. Multiple treatments with the NP combination did not induce any histological or morphological alterations in the liver, spleen, kidneys, or lungs, indicating a lack of observable side effects [130].

TAMs play a central role in maintaining the immunosuppressive TME (iTME) by releasing inhibitory cytokines such as IL-10 and TGF-β, promoting the formation of myeloid-derived suppressor cells (MDSCs), and inducing effector T-cell exhaustion [131,132,133]. TAMs account for nearly 50% of the immune infiltrate in the iTME of BM [134]. Notably, the deubiquitinating enzyme USP7 in subcutaneous Lewis cancer models is more highly expressed in M2-polarized macrophages (M2) than in M1 macrophages (M1) [134]. While USP7 inhibition has been shown to reverse the iTME in subcutaneous Lewis cancer models [134], its role in BM was specifically addressed by Lu et al. [135] They found that microparticles (MPs) released from radiation-treated tumor cells (RMPs) had a stronger capacity to activate macrophages in vitro than MPs derived from tumor cells treated with other stimuli, including chemotherapy, UV radiation, or normal culture [135]. To further enhance BBB penetration, they genetically modified RMPs to express an SR-B1-targeting peptide (R4F) on their surface, leveraging the SR-B1 receptor’s expression on BBB endothelial cells, M2/microglia, and LLC cells [135]. These engineered RMPs were then loaded with the USP7 inhibitor, P5091, generating P5091@RMPs-R4F [135]. In vitro as well as in vivo, P5091@RMPs-R4F, compared to unmodified RMPs, demonstrated significantly enhanced BBB penetration, selectively targeted F4/80+CD206+ M2/microglial cells, and reduced CD206 expression [135]. Intravenous administration of P5091@RMPs-R4F compared to an equal dose of intraperitoneal injected P5091 reprogrammed M2 by inhibiting USP7 activity and activating the MAPK signaling pathway, thereby remodeling the iTME and improving survival in LLC BM-bearing mice [135]. When combined with immune checkpoint blockade therapy, P5091@RMPs-R4F further increased effector T-cell infiltration and significantly prolonged survival [135].

Mu et al. [136] developed a novel and effective strategy to modulate the brain–tumor barrier (BTB) by co-delivering the WNT signaling inhibitor nitazoxanide (NTZ) and the BMX (bone marrow tyrosine kinase X-linked) inhibitor ibrutinib (IBR) using ICAM-1-targeted NP (NI@I-NP). This dual-targeting approach specifically suppressed WNT signaling in endothelial cells and BMX expression in pericytes within the BTB, reducing TJ integrity and increasing paracellular permeability [136]. In vivo, co-administration of NI@I-NP with either doxorubicin (Dox) or etoposide (ETO) in a mouse model significantly improved therapeutic efficacy, as both Dox and ETO were able to specifically penetrate the BTB and efficiently accumulate in BM [136]. Mice treated with Dox+NI@I-NP showed a median survival of 49 days (*p* < 0.0001 vs. free Dox; *p* = 0.0005 vs. NI@I-NP alone), while the ETO+NI@I-NP group had a median survival of 46.5 days (*p* = 0.0002 vs. free ETO; *p* = 0.0005 vs. NI@I-NP alone) [136]. Further details on these NPs are given in Table 6.

## 5. Overview of Clinical Trials

We conducted a search of three major clinical trial registries, ClinicalTrials.gov, the EU Clinical Trials Register, and the WHO ICTRP, to identify ongoing or completed trials utilizing NPs for BM. Among the NP platforms identified, AGuIX (a gadolinium-based nanoparticle) and nal-IRI (nanoliposomal irinotecan) were the most frequently studied. However, the overall number of trials remains limited, highlighting a critical need for expanded research and clinical development in the field of nanomedicine for BM. We found two clinical trials with published results. In a phase 1 clinical trial (**NCT02820454**), administration of AGuIX NP at the highest tested dose of 100 mg/kg resulted in sustained contrast enhancement of all BM larger than 1 cm in diameter for up to 7 days on MRI. This prolonged signal enhancement (SE) confirms the selective accumulation and delayed clearance of NPs within metastatic lesions. Such targeted retention is advantageous for both diagnostic imaging and radiosensitization. Importantly, to minimize toxicity and enhance treatment specificity, it is critical that NPs do not accumulate in healthy brain tissue. In this clinical study, no contrast enhancement was observed in metastasis-free regions of the brain as early as 2 h after administration, even at the highest dose, indicating favorable biodistribution and safety of AGuIX NPs. Most of the adverse events (AEs) were grade 1 and 2. Based on these promising results, phase II clinical trials are ongoing: **NANORAD 2** (**NCT03818386**) and **NANOSTEREO** (**NCT04094077**) (see Table 7). Another clinical trial, NCT01770353, conducted as a phase I study, investigated the use of Ferumoxytol (FMX) followed by MM-398 (liposomal irinotecan) for patients with cancers, including BM. In the pilot phase, researchers measured intratumoral levels of irinotecan and its active metabolite SN-38 on Day 4 of Cycle 1. The expansion phase evaluated the impact of FMX-MRI scan quality on tumor assessment and overall response. The objective response rate (ORR) in the CNS cohort was 30%, with 30% of patients achieving partial response, 30% stable disease, 20% progressive disease, and 20% not evaluable. Although grade 3 adverse events were reported.

## 6. Discussion

While the treatment paradigm for BM includes surgical resection, SRS, and, to a lesser extent, WBRT, systemic therapeutic (ST) options are rapidly evolving [15,16]. However, the BBB and the blood–CSF barrier significantly limit the ability of ST to reach therapeutic concentrations within the brain [17,18]. Most systemic agents are also non-specific and associated with systemic toxicities, posing a major challenge to achieving effective intratumoral concentrations [20]. NPs offer a highly promising solution to overcome these barriers and challenges [19,21,22]. They are often functionalized with targeting moieties that bind to specific surface proteins on the endothelial cells, facilitating NP delivery across the BBB. For example, transferrin has been used as a targeting ligand due to its affinity for the transferrin receptor (TfR) expressed on the BBB endothelium, enabling receptor-mediated transport [78]. Similarly, other ligands such as ferritin [76], PS-80 [77], CSKC peptide [86], Angiopep-2 [62], TTP [60], K-s-A [93], T12 [100], R4F peptide [135], ICAM-1-targeting γ3 peptide [136], and lexiscan [26] have been utilized to target BBB endothelial receptors, including TfR, LDLR, IGF-1R, LRP1, heparan sulfate proteoglycans (HSPGs), scavenger receptor class B type 1 (SR-B1), intercellular adhesion molecule 1 (ICAM-1), and adenosine receptors, respectively. Similarly, focused ultrasound (FUS) has been utilized to enhance NP delivery across the BBB [139,140,141,142,143]. Ngamcherdtrakul et al. demonstrated improved survival in mice following treatment with T-siHER2-NP in combination with microbubble-assisted FUS [115]. To further minimize off-target toxicities and improve tumor specificity, NPs are often functionalized with ligands that selectively bind to receptors overexpressed on metastatic cells. For instance, conjugation of anti-HER2 antibodies to NP enables targeted delivery to HER2-positive metastatic cells within the brain parenchyma while sparing healthy brain tissue [55].

From this review, it is evident that NPs can carry a range of therapeutics, including chemotherapy, immunotherapy, small molecule inhibitors, gene therapies, radiosensitizers, and TME modulators, to the BM. Additionally, NPs have shown the capability to carry multiple therapeutic agents simultaneously, facilitating combination therapy across the BBB to the BM. Numerous NP-based therapeutic delivery platforms have received FDA approval or are currently undergoing clinical trials for various brain-metastasizing cancers, including lung cancer, breast cancer, and melanoma [144,145,146,147]. However, to date, no NP-based therapies have been approved specifically for the treatment of BM, and a very limited number of ongoing clinical trials are available, creating a translational gap. Moreover, preclinical studies using NPs for the treatment of BM have shown no major complications in animal models. To advance NP-based therapies for BM, a comprehensive evaluation of their pharmacokinetics, pharmacodynamics, and safety profile is essential through well-designed clinical trials. A major limitation in the current literature is the lack of quantitative assessment of intratumoral drug concentrations following treatment, which restricts our understanding of therapeutic delivery and efficacy. Additionally, tumor volume reduction, a key indicator of treatment response, is not consistently measured or reported across studies. Furthermore, survival outcomes are either incompletely calculated or entirely omitted by some authors, limiting the ability to evaluate long-term clinical benefit and compare treatment modalities effectively. These gaps underscore the need for more rigorous and standardized reporting in future research.

Clinical trials investigating NP-based therapies for BM have primarily focused on AGuIX NPs, which act as radiosensitizers. Additionally, two clinical trials, **NCT01770353** (phase I) and **NCT03328884** (phase II), have evaluated liposomal irinotecan (Nal-IRI). Notably, all of these studies involve NPs carrying a single therapeutic agent. Currently, there are no ongoing clinical trials assessing NP-mediated delivery of other classes of therapeutics, such as chemotherapeutics beyond irinotecan, immunotherapies (e.g., TRZ), small-molecule inhibitors (e.g., lapatinib, afatinib), or gene therapies, in patients with BM. This underscores a significant gap and highlights the urgent need for clinical trials evaluating NP platforms designed to deliver a broader range of therapeutic agents, as well as the potential of combination payload strategies that have shown promise in preclinical studies.

## 7. Limitations

One of the primary challenges in advancing NP therapies for BM is the limited number of clinical trials. While preclinical research has shown promising results in vitro and in vivo small animal models, very few NPs have reached clinical application for BM patients. This disparity is largely due to a translational gap between animal and human studies, driven by an incomplete understanding of physiological and pathological differences between species, particularly regarding how these variations affect NP behavior and efficacy in vivo [148]. Additionally, inter-patient [31] and intra-patient [149] heterogeneity further complicates clinical translation, as there is a lack of studies examining how NPs interact with diverse or stratified patient populations [31]. Another major challenge in translating nanomaterials into clinical applications lies in ensuring their safety, scalable manufacturing under good manufacturing practice (GMP), and effective targeting of specific tissues [150]. Addressing these gaps is crucial for the successful development and personalization of nanomedicine in BM therapy [31].

The clinical translation of NPs could be further hindered by multiple physical and biological barriers, such as shear forces, protein adsorption, and rapid systemic clearance, which limit the proportion of administered NPs that reaches its intended therapeutic site [29]. Following systemic administration, NPs must compete with the mononuclear phagocytic system (MPS) and renal clearance pathways before reaching the tumor [151]. The MPS, comprising organs such as the liver and spleen, contains phagocytic cells that readily uptake NPs, while the kidneys rapidly excrete NPs with a hydrodynamic diameter smaller than 5.5 nm [151]. A literature review conducted by Wilhelm et al. [151] covering studies from 2005 to 2015 reported that, on average, only 0.7% of the injected NP dose is delivered to solid tumors in a mouse model. Similarly, ligand-functionalized NPs designed to cross the BBB typically achieve less than 1% accumulation in brain tissue following systemic administration [91,92,93]. One contributing factor is the body’s immune recognition of synthetic components; for example, PEGylated NPs can trigger the production of anti-PEG antibodies, leading to accelerated blood clearance upon exposure [152,153]. Moreover, once NPs enter circulation, they interact with blood components, leading to non-specific adsorption of serum proteins and lipids, forming a protein corona [154]. This corona influences the biodistribution, targeting efficiency, and stability of both the NP and its therapeutic payload [155,156]. As an example, PS-80-coated NPs may adsorb Apo-E from plasma, which facilitates BBB penetration via LDLR-mediated transcytosis [53] but can also promote hepatic uptake, leading to premature clearance from the bloodstream.

## 8. Clinical Perspectives

To overcome the persistent barriers in NP development for BM, future efforts must prioritize expanded clinical trials. Despite significant preclinical progress, the clinical application of NP remains limited, in part due to insufficient human data. Accelerating first-in-human and phase I/II trials will be essential to evaluate the safety, biodistribution, and therapeutic potential of emerging nanomedicines for BM. In parallel, incorporating biomarker-driven patient stratification can help address inter- and intra-patient heterogeneity by tailoring NP therapies to specific molecular or immune profiles, thereby enhancing therapeutic efficacy and minimizing off-target effects. Furthermore, future studies should investigate localized delivery methods, such as intrathecal, intra-surgical, or intra-tumoral delivery, which may enable NPs to bypass systemic clearance mechanisms and achieve higher concentrations at the tumor site. These localized approaches could also help circumvent challenges associated with immune recognition, protein corona formation, renal clearance, MPS, and non-specific cellular uptake. Ultimately, advancing nanomedicine for BM will require a multidisciplinary approach combining innovative materials science, translational research, clinical trial design, and personalized medicine strategies. However, the future incorporation of artificial intelligence (AI) has the potential to revolutionize the field of nanomedicine for BM. AI can facilitate patient-specific nanomedicine design by analyzing complex biological data and identifying optimal therapeutic strategies specific to individual patients. Moreover, AI-driven tools can accelerate research by optimizing NP development, predicting treatment responses, and streamlining clinical decision-making, thereby making the entire process faster, more efficient, and more precise. Bridging the translational gap between preclinical success and clinical efficacy will be critical to realizing the full potential of NPs in the treatment of BM.

## 9. Conclusions

The transformative potential of nanotechnology in the treatment of BM heralds a new era in personalized medicine, where therapies are meticulously tailored to the unique genetic and molecular profiles of each patient. This innovative approach enhances diagnostic accuracy and offers previously unattainable options for managing tumors that are difficult to access through conventional means. By precisely targeting and effectively eliminating residual tumor cells that often contribute to recurrence, nanotechnology paves the way for improved long-term outcomes and a significant reduction in the likelihood of recurrence. Hence, the promise of nanotechnology is a ray of hope in the ongoing fight against BM, as it has the potential to completely change treatment methods and eventually improve patient care as science advances. We expect nanomaterials to surpass current cancer therapy, which includes operative tumor removal, chemotherapy, and radiation therapy, in the future. The innovative use of nanocarriers in BM therapy represents a significant advancement in the fight against this complex disease. By protecting drugs from breaking down too soon and stopping them from reacting negatively with the body, these carriers improve how well the treatments work. Furthermore, their ability to control drug movement and distribution ensures that treatments reach the intended sites with precision, minimizing systemic side effects. Additionally, nanocarriers help drugs get absorbed better and enter the right cells or tissues, which increases the chances of successful treatment. Collectively, these attributes underscore the transformative potential of nanocarrier technology, paving the way for more effective and targeted cancer therapies that hold promise for improving patient prognosis and quality of life. The potential toxicity issues associated with the use of nanomaterials in patient clinical care present an important opportunity for careful consideration and innovation. By prioritizing the engineering of nanomaterials to be both biocompatible and biodegradable, we can enhance patient safety and efficacy. Moreover, it is essential to minimize accumulation in off-target organs and improve clearance rates to ensure minimal harm during application. Nanomaterials provide a diverse array of possibilities for the production of NP. However, to be truly effective, we should focus on creating formulations that are batch-to-batch consistent, easy to assemble, and able to pass rigorous clinical testing to demonstrate their specificity and safety. Lastly, a thorough evaluation of the environmental impact of the widespread use of nanomaterials will be crucial to promoting sustainable practices in this field. By addressing these considerations, we can harness the full potential of nanomaterials as a promising, effective treatment for BM. With more collaborative efforts bridging engineering, oncology, and neurosurgery, there is strong potential to turn these platforms into clinically actionable therapies. The future of nanomedicine in BM treatment lies in not only enhancing delivery but also in translating innovation into impact.

## Figures and Tables

**Table 1:** Overview of nanoparticles carrying chemotherapeutics to brain metastases.

S.No.	Nanomaterial	Structure	Targeting Moiety	Molecular/Cellular Target	Cargo	Mechanism of Action	Animal Model/Cell Line	Outcomes	Adverse Events	Reference
1	LANP	PEG Poly(ε-caprolactone) (PCL) RLQLKL	Lexiscan AMD3100-conjugate	Adenosine receptor/BBB endothelium CXCR4/tumor cells	Dox	LANP are activated by NE, allowing them to shrink in size. Lexiscan helps them pass the BBB, and AMD3100 target the tumor specifically via binding to CXCR4.	Female athymic NCr-nu/nu mice MDA-MB-231-Br-HER2 (231BR) cells	LANP conc. in BCBM was over 2.5 times higher than peripheral organs. Prolonged survival in mice.	None	[26]
2	PMAA-PS 80-g-St terpolymer (PPT)	PMAA PS-80 Starch	PS 80-bound Apo-E	LDL-R/BBB/tumor cells	Dox	PS 80 adsorb Apo-E from plasma onto the surface of NPs facilitating the NP uptake by BCECs and tumor cells through interaction with LDL-R.	immunodeficient female SCID mice. DA-MB-231-luc-D3H2LN triple-negative human BC cell line.	Dox was detected in mice brain treated with Dox-loaded NPs but not in mice treated with free Dox. Enhanced tumor cells apoptosis in NP-treated mice	None	[53]
3	iRGD-PPT	PMAA PS-80 Starch iRGD	PS 80-bound Apo-E iRGD	LDL-R/BBB/tumor cells/TAM Integrin/tumor cells	Dox & MMC	PS 80 adsorb Apo-E from plasma onto the surface of NPs facilitating the NP uptake via LDL-R by BCECs and helping them pass through BBB as well as by TAM. iRGD binds to αv integrins on the surface of BM, facilitating the NP uptake by BM cells.	NRG mice Human MDA-MB 231-luc-D3H2LN and MDA-MB-468 cells RAW 264.7 macrophages	Enhanced BBB penetration & greater drug conc. in BM. TAM Reduction in brains of NP treated BM-bearing mice on histological analysis Reduced BM progression and improved survival in mice, in vivo.	None	[54]
4	MM-302	Liposome PEG Anti-HER2 antibody	Anti-HER2 antibody	HER2-overexpressing cells	Dox	Tumor priming with CPA reduces tumor cell density and interstitial fluid pressure, increasing vascular perfusion and enhancing MM-302 delivery and penetration. Anti-HER2 antibodies selectively binds to HER-2 overexpressing on BM cells	NCR/nu and nu/nu nude mice BT474-M3/SUM190/NCI-N87/MDA-MB-361	2–3× increase in tumor drug delivery 12-fold increase in dox-positive nuclei improved tumor growth inhibition with combined CPA (synergistic), no increased deposition in heart or skin.	None	[55]
5	PRINT-C2-docetaxel	PLGA	None specified	Acidic TME (pH-triggered activation)	C2-DTX (prodrug of DTX)	NPs accumulate in tumors; prodrug converts to active DTX in acidic TME, enhancing delivery and minimizing systemic toxicity.	Foxn1 nu/nu mice A549-luc human NSCLC cell line	35% increase in median survival (90 days vs. 66.5 days with SM-DTX) 7-fold higher tumor drug accumulation compared to SM-DTX	None	[56]
6	T7-DSNP/9291	Mal-PEG-DSPE T7 peptide Dox-SS-C18 core	T7 peptide	TfR, and high GSH in tumor cells	Osimertinib (AZD9291) & Dox	BBB penetration via TfR-mediated transcytosis, GSH-triggered drug release within tumor cells for EGFR inhibition (AZD9291) and DNA damage-induced apoptosis (Dox)	Balb/c nude mice PC9/BCEC	Enhanced BBB penetration & accumulation significant tumor reduction and extended median survival (35 days vs. 21 days for control) in mice	None	[57]
7	HER3-HBP-Dox	PEGMA EGDMA hydrazone linker anti-HER3/anti-PEG bispecific abs	anti-HER3 abs	HER3 receptors/tumor cells	Dox	anti-HER3 abs to HER3 receptor crosslinking-induced endocytosis occurs by clathrin-dependent and independent endocytic pathways. hydrazone linker for acid-responsive drug release	Female NOD SCID mice BT474 and MDA-MB-231 (MDA231)	In vitro, HER3-HBP-Dox has great cytotoxicity than HER3-HBP and HBP-Dox. In vivo, HER3-HBP-Dox had great anti-tumor activity and showed prolonged survival in mice.	None	[58]
8	M@H-NP/Dox	PLGA-PLL polymer; surface-modified with PEG and hyaluronic acid (HA)	HA targets CD44 on tumor cells	CD44/BCBM KATP channels and claudin-5 on BTB endothelial cells	Minoxidil (MS) Dox	M@H-NP/Dox cross BTB via both transcellular and paracellular pathways HA targets CD44+ BM MS selectively opens BTB but spares normal BBB	BALB/c nude mice with BM via intracardiac injection 231Br (HER2+ brain-seeking breast cancer cells)-	M@H-NP/Dox enhanced Dox delivery to BM Median survival extended to 45 days vs. 28 days (saline), 38 days (free Dox) Reduced size of BM	None	[59]
9	NP (TM@TTP)/(Dox@TTP-HA; drug-loaded NP)	PLGA-PLL polymer- Surface: PEGylated and functionalized with TTP and HA	TTP HA	heparan sulfate proteoglycans (HSPGs) on BBB endothelial cells CD44/tumor cells	Tunicamycin & Dox	TTP binds to HSPGs, helping in NP uptake by BBB. HA targets CD44 on tumor TM@TTP “primes” the BBB by inhibiting Mfsd2a and enabling transcytosis Dox@TTP-HA then crosses BBB via lipid raft/caveolae and targets CD44+ metastatic cells via HA-CD44 interaction	Female BALB/c nude mice. 231Br triple-negative BCBM	Brain accumulation of Dox@TTP-HA increased 4.3-fold with TM@TTP priming Median survival: 46 days with TM@TTP + Dox@TTP-HA vs. 27 days with saline (*p* < 0.001), 33 days with free Dox (*p* < 0.001), 37 days with Dox@TTP–HA (without TM@TTP, *p* < 0.01), and 39.5 days with TM@TTP and Dox@TTP (without HA, *p* < 0.05).	None	[60]
10	hDOX@NP—dual-targeting PLGA-based NP	PLGA-PLL NPs functionalized with transcytosis-targeting peptide (TTP)	TTP HA	HSPGs/BBB endothelial cells CD44/BCBM	hDOX -a prodrug of Dox conjugated to HA	TTP-HSPGs interaction enables caveolae-mediated BBB transcytosis HA-CD44 mediated tumor cell endocytosis hDOX is activated by hyaluronidase highly expressed in BCBM.	Female BALB/c nude mice 231Br (brain-seeking HER2+ BC cells)	Median survival: 55 days with hDOX@NP (vs. 28.5 days with saline, 38 days with Dox, 43 days with Dox@NP) Improved tumor accumulation, BBB penetration, and survival Reduced Dox efflux from tumor cells	None	[61]
11	S@A-NP/Dox: Simvastatin (SIM)-loaded Angiopep-2-anchored NP Dox	PLGA anchored with Angiopep-2 peptide	Angiopep-2 peptide	LRP1 receptor on brain BMECs and tumor cells	SIM and Dox	Angiopep-2 facilitates LRP1-mediated transcytosis across BBB and endocytosis in tumor cells SIM enhances LRP1 expression, amplifying BBB crossing and tumor targeting	Cell Lines: bEND.3 (BMECs), MDA-MB-231Br (BCBM model) Animal Model: BALB/c nude mice	enhanced BBB penetration and tumor cells target Increased LRP1 expression in BMEC and tumor cells high Dox accumulation in BM & reduced proliferation Prolonged median survival in mice (59.5 days vs. 52 days with free Dox, 40 days with S@A-NP, 39.5 days with saline)	None	[62]
12	ACUPA and cyclic TT1 (cTT1) functionalized NP (A-NP-cT)	PLGA-PLL Surface: modified with ACUPA and cTT1 peptide via PEG linker	ACUPA cTT1 peptide	PSMA on BTB endothelial cells p32 on BCBM	Dox and LAP	ACUPA targets PSMA on BCBM-associated BTB endothelial cells enabling PSMA-mediated transcytosis across BTB cTT1 enables p32-mediated endocytosis into tumor cells	BALB/c nude mice 231Br (HER2+ brain-seeking breast cancer cells)-	4.57-fold higher BTB penetration than BBB Most effective tumor suppression seen with Dox/LAP combo in NPs Median survival: 44 days vs. saline (25 d), free combination (29 d), unmodified NP (29 d), A-NP (33 d), NP-cT (32 d)	None	[63]
13	TAT-Au-Dox NP	PEGylated gold HIV-derived TAT peptide (YGRKKRRQRRR)	TAT	BBB/tumor cells	Dox	TAT helps in BBB penetration Dox is bound to the surface of particles via a pH-sensitive hydrazone bond, which in TME releases Dox under low PH	athymic nude mice MDA-MB-231-Br and CN-34-Br cells	TAT-Au-Dox NPs led to significantly more Dox uptake within cancer cells compared to free Dox. Decreased survival in TAT-Au-Dox treated cell compared to Au-Dox and free Dox. improved median survival (39 days) compared to free Dox group (25 days) and PBS group (32 days)	None	[64]
14	cFd-Lip/PTX	PEGylated liposomes PTX	FA dNP2 peptide	Folate receptors/Tumor cells	PTX	Upon reaching the TME, the cleavage of FA and the subsequent exposure of the cell-penetrating peptide dNP2 can significantly enhance the internalization process within CAFs, BBB and tumor cells.	BALB/c mice 4T1 cells & NIH 3T3 cells.	In vitro studies showed that bEnd.3 cells took up cFd-Lip more than Fd-Lip, dNP2-Lip, FA-Lip, and PEG-Lip, suggesting that FA and dNP2 have synergistic effects on transmigration across the bEnd.3 monolayer. cFd-Lip/PTX significantly induced tumor apoptosis and inhibited the proliferation and improved survival in mice.	None	[65]
15	PTX-OA NP	Oleanolic acid (OA)	Oleanolic acid (OA)	Cannabinoid receptor 1 (CB1)	PTX	Hypothetically penetrate the brain through interaction with cannabinoid receptor 1 (CB1) on BBB The leaky nature of BBB in BCBM and ERP enhance accumulation.	Female athymic NCr-nu/nu mice. MDA-MB-231BR cells	After 24 h, IR780-OA NPs in mice bearing BCBM was 2× than free dye. After 18 days, the tumor volume reduced by 69% in PTX-OA NP-treated mice compared to the PBS and 15% and 14% in free PTX and OA NP groups, respectively, while 85% compared to that of the PBS group after 25 days.	None	[66]
16	PEGylated liposomal Dox (PLD) vs. Doxorubicin (NonL-Dox)	Dox encapsulated in PEGylated liposomes	None	None	Dox +ABT-888 (inhibitor of a PARP)	Passive targeting via enhanced permeability and retention effect; prolonged circulation due to PEGylation allows accumulation in TME and enhanced drug delivery across BBB	Foxn1nu/nu mice MDA-MB-231-BR cell line Female,	PLDL-Dox showed 1,500-fold higher plasma and 20-fold higher tumor AUC compared to NonL-Dox Median survival: PLD (32 days) vs. NonL-Dox treatment (23.5d [CI 18–28], *p* = 0.0002). Combination treatment with PLD/ABT-888 improved survival compared to NonL-Dox/ABT-888 (35d [CI 31–38] versus 29.5d [CI 25–34]; *p* = 0.006).	None	[67]
17	DTX-NP	PMAA PS-80 Starch	PS 80-bound Apo-E	LDL-R/BBB/tumor cells	DTX	PS 80 adsorb Apo-E from plasma onto the surface of NP. Apo-E facilitates the uptake of the NPs by BCEC and tumor cells through interaction with LDL-R family members expressed on these cells.	SCID mice MDA-MB-231-luc cells	2.7× DTX conc. in the tumor-bearing brain 15 min after treatment compared to equivalent dose of free DTX. 2× DTX conc. in the tumor-bearing brain compared to normal brain 15 min after injection of NP. In vivo, DTX-NPs delayed tumor growth by 11-fold and prolonged median survival time by 1.9-fold compared to free DTX.	None	[68]
18	Ang-MIC-PTX/LP	Micelles made of PEG-PLA Angiopep-2	Angiopep-2 peptide	LRP1	PTX and LAP	Angiopep-2 facilitates LRP1-mediated transcytosis across BBB and endocytosis in BM.	Balb/c nude mice SKBr-3 cells, 4T1 cells and BCECs	NPs crossed the BBB and specifically hit the tumor. The NPs showed a significant improvement in survival rate (56 days) compared to the saline-treated group (35 days).	None	[69]

**BCBM** = breast cancer brain metastasis, **NE** = neutrophil elastase, **LANP** = lexiscan-loaded, AMD3100-conjugated, shrinkable NPs, **PMAA** = poly(methacrylic acid), **Dox** = doxorubicin, **PS-80** = Polysorbate, **BC** = breast cancer, **Apo-E** = apolipoprotein-E, **MMC** = mitomycin C, **iRGD** = internalizing Arginine-Glycine-Aspartate peptide, **TAM** = tumor associated macrophages, **PEG** = polyethylene Glycol, **PLGA** = poly(lactic-co-glycolic acid), **TME** = tumor microenvironment, **DTX** = docetaxel, **SM-DTX** = standard small molecule DTX, **DSPE** = distearoylphosphatidylethanol-amine, **TfR** = transferrin receptor, **PEGMA** = poly(ethylene glycol) methacrylate, **EGDMA** = Ethylene glycol dimethacrylate, **PLL** = poly(ε-carbobenzoxy-l-lysine), **HA** = hyaluronic acid, **TAT** = trans-activating transcriptional activator, **CAF** = cancer associated fibroblast, **PTX** = paclitaxel, **EPR** = enhanced permeability and retention, **PARP** = Poly (ADP-ribose) polymerase, **PLA** = poly(lactic acid), **BCECs** = brain capillary endothelial cells, **CPA** = cyclophosphamide, **BTB** = blood–brain tumor barrier.

**Table 2:** Overview of nanoparticles carrying immunotherapeutic agents.

S.No.	Nanomaterial	Structure	Targeting Moiety	Molecular/Cellular Target	Cargo	Mechanism of Action	Animal Model/Cell Line	Outcomes	Adverse Events	Reference
1	H-TZ	Human ferritin NPs covalently conjugated with TRZ via PEG linker	Ferritin	TfR1/BBB	TRZ (anti-HER2 antibody)	Crosses BBB via TfR1, targets HER2+ tumor cells, enhances antibody delivery and retention, activates macrophage-mediated killing, modulates neuroinflammation	BALB/c nude mice D2F2/E2/D2F2/E2-Luc HER2+ breast cancer cells	Significant reduction in tumor growth in vivo. increased macrophage activation and anti-inflammatory cytokine in expression in-vivo better efficacy than free TZ in vivo	None	[76]
2	TRZ terpolymer NP (TRZ-TNP)	PMAA PS-80 Starch	PS 80-bound Apo-E	LDL-R/BBB/tumor cells	TRZ	PS 80-bound Apo-E-LDLR-mediated transcytosis. TRZ target HER2+ cells.	SCID mice HBEC/BT474/RBE4 cell line	4-fold higher TRZ-TNP in mice compared to normal brain. TRZ–TPN resulted 50-fold increased TRZ levels. TRZ–TPN inhibited tumor growth by 43-fold than free TRZ in mice. >1.3-fold increase in survival compared to an equivalent dose of free TRZ.	None	[77]
3	Transferrin receptor (TfR)-targeted mucic acid polymer (MAP) NP	MAP Camptothecin (CPT) conjugated to MAP Transferrin (Tf) Herceptin (TRZ) via acid-cleavable boronic acid-diol linkage	Tf	TfR/BBB HER2/tumor cells	Herceptin CPT	TfR-mediated transcytosis across the BBB pH-sensitive detachment of targeting ligands in endosome. Herceptin target HER2 CPT inhibit topoisomerase I	Rag2–/–;Il2rg–/– mice HER2+ BT474-Gluc human BCBM cell line	In vivo, combined CPT/Herceptin loaded NPs showed greatest tumor inhibition and durable response compared to either CPT or Herceptin loaded NP. Free CPT + Herceptin had minimal impact	None	[78]
4	Rituximab (RTX) NP (n-RTX) CXCL13-conjugated RTX NP (n-RTX^CXCL13)	Zwitterionic polymer shell made of 2-methacryloyloxyethyl phosphoryl-choline (MPC; monomer), PLA-PEG-PLA and/or GDMA (crosslinkers), conjugation to CXCL13	CXCL13	BBB penetration via choline/acetylcholine transporter interactions with MPC shell CXCR5/Lymphoma cells	Rituximab (anti-CD20 mAbs)	CXCL13 conjugated to the surface, enabling NP targeting to CXCR5-expressing lymphoma cells. Lower PH in tumor accelerates the release of RTX	2F7-BR44 (AIDS-associated B-cell NHL line adapted for BM) (NOD)-SCID gamma (NSG) and humanized BLT mice with CNS lymphoma	CNS RTX levels with n-RTX increased ~10-fold compared to native RTX. n-RTX significantly reduced the lymphoma burden in the brain. n-RTX^CXCL13 showed complete CNS lymphoma clearance in BLT mice. Improved survival in both NSG and BLT models	None	[74]
5	SIL@T NP	Hydrophobic poly-phenylalanine (pPhe) core encapsulating SIL Shell: PEG-pLys conjugated with OXA Surface: Functionalized with CSKC	CSKC peptide (analog of IGF-1)	IGF-1R on brain endothelial cells and metastatic cells	Silybin (SIL) and Oxaliplatin (OXA)	Crosses BBB and target tumor via IGF-1R-mediated transcytosis Redox-triggered release of SIL and OXA in tumor cells SIL -STAT3 inhibitor OXA- induce immunogenic cell death	SPF grade c57 nude mice 4T1-Br (brain-seeking murine TNBC cells)	Significant reduction in BM burden Median survival extended (more than 21 days) induced robust antitumor immune responses (increase effector T cell infiltration, reduce TAM M2 polarization and Treg cell infiltration)	None	[86]

**PMAA** = Poly(methacrylic acid), **MAP** = mucic acid polymer, **PLA** = poly(lactic acid), **PEG** = Polyethylene glycol.

**Table 3:** Overview of nanoparticles carrying small-molecule inhibitors.

S.No.	Nanomaterial	Structure	Targeting Moiety	Molecular/Cellular Target	Cargo	Mechanism of Action	Animal Model/Cell Line	Outcomes	Adverse Events	Reference
1 *	Lipid-polymeric NP	mPEG DSPE PLGA Lecithin Peptide, FD7 and CCD	FD7 and CCD peptides	TJ proteins/BBB	Afatinib	The peptides FD7 and CCD of LPN disrupt tight junction proteins, helping LNP primarily traverse the BBB via a paracellular route. They also undergo partial transcellular transport.	bEnd.3 cells PC9 cells	LPN increase BBB permeability Enhanced cytotoxicity against PC9 cells across BBB model of bEnd.3 cells No in vivo experiments done.	NA	[89]
2	NP-K-s-A	PLGA PLL MAL-PEG-SCM K-s-A fusion peptide	K-s-A	LRP1/BBB HER2/tumor cells	Lapatinib Dox	K-s-A binds to LRP1 on BBB and mediate endocytosis of NP-K-s-A. MMP1 then cleaves K-s-A into angiopep-2 and KAAYSL. Angiopep-2 inhibits LRP1, reducing NP clearance and KAAYSL, targets HER2 on tumor cells	bEND.3 cells 231Br cells	5-fold higher NP uptake in brain bearing BCBM compared to control Prolonged survival in mice bearing BCBM	None	[93]
3	T12/PD-L1-Nb-modified liposomes (T12/P-Lipo)	PEGylated liposome Anti-PD-L1 nanobody T12 peptide (TfR-binding)	Anti-PD-L1 nanobody T12 peptide (TfR-binding)	PD-L1 on TAM and tumor cells; TfR on endothelial and tumor cells	Simvastatin (SV), Gefitinib (Gef)	BBB penetration via T12-TfR interaction. TAM repolarization (M2→M1), ROS elevation, inhibition of EGFR/Akt/Erk signaling pathway, reversal of EGFR T790M-associated drug resistance.	Balb/c nude mice H1975 cells/BCEC/HUVEC	Increased ROS and apoptosis, downregulated p-EGFR/p-Akt/p-Erk prolonged median survival (29 days), reduced tumor size and proliferation	None.	[98]
4	T12 peptide-modified bovine serum albumin nanoparticles (T12-BSA NP)	<135 nm nanoparticles with negative ζ-potential, formed via albumin denaturation and conjugated with T12 peptide	T12 peptide (targets transferrin receptor), albumin (binds SPARC protein)	TfR & SPARC/BBB/TAM	Disulfiram/copper ion chelate (DSF/Cu) and regorafenib (Rego)	Penetrates blood–brain barrier via TfR-mediated transport Targets tumor and TAM via SPARC and TfR DSF inhibits FROUNT to repolarize TAM (M2 → M1) Rego inhibits VEGFR signaling and angiogenesis	Balb/c-nude mice H1975/BCEC/H1975/AZDR	Tumor inhibition: 91% in subcutaneous model Median survival extended to 36 days in BM model (vs. 30 days for non-targeted NP and <20 days for untreated) Decreased M2 macrophages and MDSCs, increased M1 macrophage population	None	[100]
5	LHNP	Core-shell made from HSA and phosphatidylcholine	Albumin	HER2/EGFR on tumor cells and P-gp/BCRP at BBB	Lapatinib (Lap)	Inhibits HER2 and EGFR signaling, albumin NP bypass P-gp efflux, increase BBB penetration, and enhance tumor accumulation via EPR and gp60/SPARC	BALB/c mice 4T1/4T1-luc cell line	5.4× higher brain accumulation of NP-based Lap than free Lap inhibition of adhesion, migration, invasion, ↓ MMP-2, uPA, OPN, CXCR4 median survival ↑ from 19.1 to 36.4 days in mice	None	[101]

* no in vivo findings were measured, **NA** = not available, **LPN** = lipid-polymeric NP, **NPs** = nanoparticles, **DSPE** = distearoylphosphatidylethanolamine, **PLGA** = poly(lactic-co-glycolic acid), **PLL** = poly(ε-carbobenzoxy-l-lysine), **MAL-PEG-SCM** = maleimide polyethylene glycol succinimidyl carboxymethyl ester, **K-s-A** = KAAYSL—VPMS-MRGG— angiopep-2, **VPMS-MRGG** = valine-proline-methionine-serine-methionine-arginine-glycine-glycine, **MMP1** = Matrix Metalloproteinase 1, **NA** = Not available, **P-gp** = P-glycoprotein, **uPA** = urokinase-type plasminogen activator, **OPN** = osteopontin, **ROS** = reactive oxygen species, **SPARC** = secreted protein acidic and rich in cysteine, **VEGFR** = vascular endothelial growth factor, **TNBC** = triple-negative breast cancer, **LHNP** = loaded human serum albumin NP, **BCRP** = breast cancer resistance protein, HSA = Human serum albumin, ↓ = decrease, ↑ = increase.

**Table 4:** Overview of nanoparticles carrying therapeutic genes.

S.No.	Nanomaterial	Structure	Targeting Moiety	Molecular/Cellular Target	Cargo	Mechanism of Action	Animal Model/Cell Line	Outcomes	Adverse Events	Reference
1	T-M/siRNA micelle (CSKC-PEG-pArg-pLys-SS-PTX)	CSKC peptide PEG Disulfide bond Polyarginine Polylysine	CSKC peptide	IGF-1R/BBB/tumor cells	PCDH 7-targeting siRNA Paclitaxel	CSKC peptide of NPs bind to IGF-1R on the surface of BBB`s endothelial and tumor cells helping NP passing BBB and targeting tumor cells. The micelles release the free drugs PTX and siRNA in a high GSH environment of tumor. siRNA targets PCDH 7 of GJs, disrupting communication between astrocytes and BM. PTX targets BM.	Nude mice MDA-MB-231/Luc	Enhanced cellular uptake Decreased expression of PCDH 7 and GJs between BM cells and astrocytes Enhanced in vitro cytotoxicity Improved survival in treated mice	None	[105]
2	Modified MN (MN-anti–miR-10b)	Magnetic NP Near infrared dye (Cy5.5-NHS) Linker SPDP	cyclic RGDfK (cRGD)	αvβ3/αvβ5 integrins/BBB/tumor cells	anti–miR-10b	cRGD-integrin mediated endocytosis of MN-anti–miR-10b. anti–miR-10b targets upregulated miRNA-10b in BM, disrupting its role in BM.	Balb/c nude mice MDA-MB-231-BrM2-831 cells	NPs cross the BBB and accumulate in BM. BM-bearing mice treated with MN-anti–miR-10b showed marked regression of tumor lesion compared to control group treated with only MN.	None	[109]
3	Engineered exosome (Exo^scFv^)	Same as exosome Lipid protein	anti-EGFR scFv-lamp2b	EGFR/tumor cells	LPCAT1-targeting siRNA	Exosomes are biological NP, so they passively cross the BBB. But the anti-EGFR scFv-lamp2b selectively bind to EGFR on the surface of BM.	BALB/C nude mice HEK293T cells and PC9 cells	Reduced BM burden in mice treated	Weight loss in treated mice	[114]
4	T-siHER2-NP(DTX)	PEI MSNP PEG TRZ	TRZ	HER2 Receptors	Docetaxel (DTX) HER2 siRNA TRZ	Focused ultrasound-based BBB penetration of NP. TRZ targets HER2 on tumor cells. siRNA inhibits the synthesis of HER2 protein.	SCID female mice BT474/HCC1954/MCF7 cell lines LM2-4luc+H2N/BTGFL1 cell lines	The majority of the HER2+ cell line (BT474) underwent apoptotic death. Mice treated with MB-FUS plus T-siHER2-NP(DTX) showed inhibition of intracranial BTGFL1 tumor growth.	None	[115]
5	AP30NP Poly(lactone-*co*-β-amino ester)	PDL MDEA TDDP AMD3100 PEG	AMD3100	CXCR4/tumor cells	proMel gene	AMD3100-CXCR4 interaction based tumor target. proMel Gene synthesize secretory promelittin, which is converted to melittin by MMP in TME. Melittin is tumor cytolytic.	Female nude mice (NCr nu/nu) NHA/231BR cells	significantly inhibited tumor progression in vivo. increased cellular apoptosis in tumors. No significant damage to normal brain tissue around tumors.	None	[118]

**CSKC** = cysteine-serine-lysine-cysteine, **IGF-1R** = insulin-like growth factor 1 receptor, **PEG** = polyethylene glycol, **GSH** = glutathione, **GJs** = gap junctions, **SPDP** = N-succinimidyl 3-[2-pyridyldithio]-propionate, **MN** = magnetic nanoparticle, **RGDfK** = Arg-Gly-Asp-D-Phe-Lys, **EGFR** = epidermal growth factor receptor, **MSNP** = Mesoporous silica NP, **PEI** = polyethyleneimine, **TRZ** = trastuzumab, **MB-FUS** = microbubble-assisted focused ultrasound, **PDL** = ω-pentadecalactone, **MDEA** = *N*-methyldiethanolamine, **TDDP** = diethyl 3,3′-(4,4′-trimethylenedipiperidine-1,1′-diyl) dipropionate.

**Table 5:** Overview of nanoparticles carrying radiotherapeutic/radiosensitizer.

S.No.	Nanomaterial	Structure	Targeting Moiety	Molecular/Cellular Target	Cargo	Mechanism of Action	Animal Model/Cell Line	Outcomes	Adverse Events	Reference
1	AGuIX	Polysiloxane Gd-oxide	None	BBB/Tumor cells	Gd	AGuIX accumulate passively and preferentially in brain tumors due to blood–brain barrier is damaged	C57BL/6J mice B16F10 mouse melanoma cell line	No invitro cytotoxicity of AGuIX Enhanced cytotoxicity by AGuIX & radiation Prolonged survival in mice	None	[119]
2	Iodine NP (INP)	polymer of triiodo-benzene PEG	None	Tumor cells (via EPR effect)	Iodine	Passive targeting via EPR effect. Iodine absorbs X-rays during RT, effectively boosting RT dose at the tumor, creating free radicals leading to increased DNA damage in cancer cells.	Athymic nude mice MDA-MB-231 human TNBC cell line	Median survival extended from 61 to 85 days Long-term remission: 40% of mice surviving 150 days and 30% surviving >280 days	None	[120]
3	LRRC31 NP	HDL-DES-MDEA terpolymers chlorotoxin (CTX) encapsulation of lexiscan	Lexiscan CTX	AR/BBB Tumor cells	LRRC31 DNA plasmid	Lexiscan helps NPs pass the BBB, and CTX enables them to target the tumor cells specifically. LRRC31inhibits DNA DSB repair sensitizing tumor cells to radiation.	mice (BALB/c nu/nu HEK293, MCF7, 231BR, and 4T1-BR5 cell lines	significantly inhibited tumor growth; increased apoptosis and decreased proliferation. prolonged survival in treated mice.	None	[121]

**ABTT NP** = autocatalytic brain tumor-targeted nanoparticles, **HDL-DES-MDEA terpolymers**—**HDL** = 16-hexadecanolide, **DES** = diethyl sebacate and **MDEA** = *N*-methyldiethanolamine, **AR** = adenosine receptor, **DSB** = double-strand break.

**Table 6:** Overview of the nanoparticles carrying tumor microenvironment modulators.

S.No.	Nanomaterial	Structure	Targeting Moiety	Molecular/Cellular Target	Cargo	Mechanism of Action	Animal Model/Cell Line	Outcomes	Adverse Events	Reference
1	Lasomes	Liposome CM of RA CM of BM cell	Portion of CM of RA and/or BM cells	CM of RA and/or BM cells	carbenoxolone	Las, being biomimetic, use homotypic CM recognition mechanism to carry CBX to astrocytes and BM cells, blocking GJs between them, enhancing DNP chemosensitivity.	C57BL/6 mice LLC-BrM cell line	Improved survival in treated mice Decreased tumor progression in treated group Favorable biocompatibility and biosafety.	None	[127]
2	P5091@RMPs-R4F	Same as microvesicles R4F peptide	R4F peptide	SR-B1 receptor/BBB/TAM	P5091 (USP7 inhibitor)	R4F peptide binds SR-B1 receptor, mediating P5091@RMPs-R4F cross the BBB and targeting M2. P5091 reverses immune-suppressive TME.	C57BL/6 female mice bEND.3 cells LLC Cell Line BV2 cells	Increased BBB crossing in vitro as well as in vivo. Prolonged survival in mice.	NS	[135]
3	R&B/NP	PLGA core coated with erythrocyte and MDA-MB-231Br hybrid cell membranes	Hybrid membrane (erythrocyte + 231Br cell membrane)	CM of Inflammatory BBB/tumor cells	Dexamethasone (Dex), Embelin (Emb)	Hybrid membrane, being biomimetic, use homotypic CM recognition to target the BBB and tumor cells. Inhibits secretion of serpin B2 and neuroserpin to restore plasmin activity, plasmin cleaves L1CAM and converts FasL to sFasL, leading to apoptosis and blocking vessel-associated spread	BALB/c nude mice MDA-MB-231Br cells/bEND.3/RAW264.7 macrophages/BV2 microglia/HT22 neurons	Significantly reduced intracranial tumor growth prolonged survival (median 46.5 vs. 26 days) increased apoptosis and decreased L1CAM expression	None	[130]
4	NI@I-NP: Physical combination of two ICAM-1-targeted NP: NTZ@I-NP and IBR@I-NP	PLGA-PLL NP functionalized with ICAM-1-targeting γ3 peptide via PEGylation	ICAM-1-targeting γ3 peptide	ICAM-1 on BTB endothelial cells and tumor pericytes	NTZ@I-NP loaded with Nitazoxanide I@I-NP loaded with Ibrutinib	NP selectively binds to ICAM-1 on BTB Nitazoxanide inhibits WNT signaling resulting TJs opening Ibrutinib inhibit BMX, depleting neoplastic pericyte to synergistically and specifically open the BTB	BALB/c nude mice 231Br (HER2+ brain-seeking breast cancer cells)	Sequential drug release of NTZ then IBR opens BTB Median survival improved: 49 days with Dox+NI@I-NP (compared to free Dox, *p* < 0.0001 and NI@I-NP, *p* = 0.0005) and 46.5 days with ETO+NI@I-NP (vs. free ETO, *p* = 0.0002 and NI@I-NP, *p* = 0.0005)	None	[136]

**CM** = cell membrane, **RA** = reactive astrocytes, **BM cells** = brain metastatic cells, **LAs** = LAsomes, **GJs** = gap junctions, **CBX** = carbenoxolone, **DNP** = docetaxel-loaded human serum albumin nanoparticles, **LLC** = **Lewis lung carcinoma, BV2 cells = murine (mouse-derived) microglial cell line, USP7 (ubiquitin-specific protease 7), TAM = tumor associated macrophages, NS = not specific, PLGA** = poly(lactic-co-glycolic acid), **BMX** = bone marrow and X-linked nonreceptor tyrosine kinase, **BTB** = blood–tumor barrier.

**Table 7:** Overview of the clinical trials of nanoparticles for brain metastases.

ID	Country	Phase	Status	Nanoparticle	Therapeutic	Mechanism	Primary Outcome	Published Results	Adverse Events (AEs)	PI	Reference to Results
**NCT02820454**	France	I	Completed	AGuIX	Gd/WBRT	Passively cross the BBB Radiosensitize BM cells	To determine the maximum tolerated dose (MTD)	MTD = 100 mg/kg linear relationship between the MRI SE and the [NP] MRI SE in BMs sustained for upto 7 days. No MRI SE in BM-free brain tissue AGuIX likely sensitizes BM to radiation in a dose–response manner.	Mostly, grade 1 & 2 AEs at 100 mg/kg	Camille VERRY, MD	[137]
**NCT03818386**	France	II	Active, not recruiting	AGuIX	Gd/WBRT	Passively cross the BBB Radiosensitize BM cells	Evaluation of BM response, according to RECIST v1.1 criteria (or modified RECIST) by MRI at 6th week and 3rd month	NA	NA	Camille VERRY, MD	NA
**NCT04094077**	France	II	Terminated	AGuIX	Gd/stereotactic radiation	Passively cross the BBB Radiosensitize BM cells	Rate of local control of BM	NA	NA	NA	NA
**NCT04899908**	US	II	Recruiting	AGuIX	Gd/stereotactic radiation	Passively cross the BBB Radiosensitize BM cells	Local Recurrence (RANO criteria)	NA	NA	Ayal Aizer, MD, MHS	NA
**NCT03328884**	Spain	II	Completed	Nal-IRI	Irinotecan/naI-IRI monotherapy	Biomimetic crossing of BBB by NP TPI inhibition	Efficacy in term of overall response rate	NA	NA	Javier Cortes	NA
**EUCTR2018-003994-80-FR**	France	II	unknown	AGuIX	Gd/stereotactic radiation	Passively cross the BBB Radiosensitize BM cells	rate of local control	NA	NA	Ronan TANGUY, MD	NA
**NCT05255666**	US	II	withdrawn	Nal-IRI	Nal-IRI/Pembrolizumab	Biomimetic crossing of BBB by NP TPI & PD-1 inhibition	CNS disease control rate (RANO) for 6 months	NA	NA	Ashley Frith, MD	NA
**NCT01770353**	US	I	Completed	Ferumoxytol/MM-398 (liposomal Irinotecan)	Ferumoxytol followed by MM-398	Biomimetic crossing of BBB by NP	Pilot Phase: Tumor Levels of Irinotecan and SN-38 at Cycle 1 Day 4 Expansion Phase: Impact of the Quality of MRI Scan on Tumor Evaluation & Overall Tumor Response	ORR for the CNS cohort was 30.0% PR = 30%, SD = 30%, PD = 20%, NE = 20%	Grade 3 AEs	NA	[138]

**Gd** = gadolinium, **BM** = brain metastasis, **WBRT** = whole brain radiation therapy, **NA** = not available, **RANO** = Assessed with Response Assessment in Neuro-Oncology, **[NP]** = concentration of nanoparticles, **nal-IRI** = irinotecan-load nanoliposome, **AGuIX** = activation and guidance of irradiation by X-ray, **TPI** = topoisomerase I, **PD-1** = programmed death-1, **ORR** = objective response rate, **PR** = partial response, **SD** = stable disease, **PD** = progressive disease, **NE** = not evaluable.
